# Humans significantly metabolize and excrete the mycotoxin deoxynivalenol and its modified form deoxynivalenol-3-glucoside within 24 hours

**DOI:** 10.1038/s41598-018-23526-9

**Published:** 2018-03-27

**Authors:** Arnau Vidal, Liesel Claeys, Marcel Mengelers, Valérie Vanhoorne, Chris Vervaet, Bart Huybrechts, Sarah De Saeger, Marthe De Boevre

**Affiliations:** 1 Laboratory of Food Analysis, Department of Bioanalysis, Faculty of Pharmaceutical Sciences, Ghent University, Ghent, Belgium; 2 National Institute of Public Health and the Environment, Department of Food Safety, Bilthoven, The Netherlands; 3 Laboratory of Pharmaceutical Technology, Department of Pharmaceutics, Faculty of Pharmaceutical Sciences, Ghent University, Ghent, Belgium; 4 Veterinary and Agrochemical Research Centre, Tervuren, Brussels, Belgium

## Abstract

For the first time, a comprehensive human intervention study was conducted to unravel the urinary excretion profile and metabolism of the fungal metabolite deoxynivalenol (DON) and its modified form deoxynivalenol-3-glucoside (DON-3-glucoside). Twenty volunteers were restricted in consuming cereals and cereal-based foods for 4 days. At day 3, a single bolus of 1 µg/kg body weight of DON and a single bolus of 1 µg/kg body weight of DON-3-glucoside after a washing-out period of two months was administered, and a 24-h urine collection was performed. The urine was analysed for DON, DON-3-glucoside, 3-ADON, 15-ADON, deepoxy-deoxynivalenol (DOM-1), deoxynivalenol-3-glucuronide (DON-3-glucuronide) and deoxynivalenol-15-glucuronide (DON-15-glucuronide). The urinary biomarker-analysis revealed that DON and DON-3-glucoside were rapidly absorbed, distributed, metabolized and excreted. Sixty-four % of the administered DON and 58% of DON-3-glucoside was recovered in the urine collected within 24 h. DON-15-glucuronide was the most prominent urinary biomarker followed by free DON and DON-3-glucuronide. Moreover, correlations among the presence of DON-15-glucuronide and DON-3-glucuronide were observed (within 24 hours (r = 0.61)). The DOM-1 detected in the urine was higher after the DON-3-glucoside administration. The obtained results are imperative to construct a standardized method to estimate DON-intake by means of urinary biomarkers.

## Introduction

Deoxynivalenol (DON), also known as vomitoxin, is a foodborne mycotoxin that acts as a potent inhibitor of protein synthesis. It stimulates the pro-inflammatory response, causes ribotoxic stress, cytotoxicity and apoptosis, and results in the impairment of multiple physiological functions, such as the intestinal barrier, growth, immune regulation and reproduction^1–3^. Furthermore, this fungal toxin has been linked to acute animal and human gastro-enteritis outbreaks^4^.

DON is a common mycotoxin in cereals and cereal-based food products^5,6^. The most important contributors to chronic dietary exposure to DON are bread, rolls, fine bakery wares and pasta^7^. Additionally, the mean chronic dietary exposure is above the group tolerable daily intake (group-TDI) of 1 µg/kg body weight/day (DON, deoxynivalenol-3-glucoside (DON-3-glucoside), 3-acetyldeoxynivalenol (3-ADON) and 15-acetyldeoxynivalenol (15-ADON)) in infants, toddlers and other children, and at high exposure also in adolescents and adults in Europe^8,9^. These facts indicate a potential health concern^7–9^.

Free mycotoxins, such as DON, are, however, not the only hazard in our foods. Modified mycotoxins are free toxins conjugated to more polar functionalities such as glycosyl and sulfate residues, or attached to polymeric carbohydrates or protein matrices^10,11^. The modified forms may originate from plants, fungi, mammals and/or food processing. A major concern and potential risk for consumers is the possible hydrolysis of these modified mycotoxins into their toxic free forms during mammalian digestion^12–14^. The co-occurrence of free and modified DON forms has been documented in raw wheat, especially DON-3-glucoside, 3-ADON and 15-ADON. Reported levels of DON-3-glucoside are variable, however, the concentration of DON-3-glucoside can be considered high, and even similar to DON in processed cereals^15^. 3-ADON and 15-ADON have also been detected in cereals and cereal-based products, frequently in a lower incidence than DON-3-glucoside^15,16^.

Berthiller *et al*. (2011) demonstrated that several lactic acid bacteria hydrolyse DON-3-glucoside *in vitro*, which has been a first step to prove the toxicological relevance of DON-3-glucoside^17^. On the other hand, 3-ADON and 15-ADON are rapidly converted to DON during digestion^12,18^. Thus, due to the high presence of DON modified forms in food, and the presumable transformation to DON, the FAO/WHO Joint Expert Committee on Food Additives and Contaminants (JECFA) considered 3-ADON, 15-ADON and DON-3-glucoside to be an additional contributing factor of the total dietary DON-exposure^19^.

Recently, EFSA reported on the risks to human and animal health related to the presence of DON and its acetylated and modified forms in food and feed^7^. Exposure estimates derived from the biomarker data from three European countries were of the same order of magnitude as the exposure estimates, through the deterministic probabilistic approach, for the sum of DON, 3-ADON, 15-ADON and DON-3-glucoside. The estimates of mean acute exposure to the sum of these mycotoxins across 39 different dietary surveys and all age groups using the minimum lower bound and maximum upper bound concentrations ranged from 0.2 to 2.9 µg/kg body weight/day.

Based on all the available scientific data a group-TDI (DON, 3-ADON, 15-ADON and DON-3-glucoside) of 1 µg/kg body weight/ day was established.

To fully understand DON exposure, the analysis of urinary levels of DON has been proposed due to its short excretion half-life. A few studies revealed that DON-glucuronides, which are the main phase II metabolites of DON, are the most common biomarkers in urine, especially deoxynivalenol-3-glucuronide (DON-3-glucuronide) and deoxynivalenol-15-glucuronide (DON-15-glucuronide)^20^. The analysis of urinary glucuronides is crucial for the study of trichothecene biomarkers, because approximately 90% of DON excreted via urine is conjugated with glucuronic acid^7^. To determine the urinary glucuronides, a preliminary approach was developed based on the enzymatic hydrolysis of deoxynivalenol-glucuronides, and subsequent determination of the “total DON” (sum of free and released mycotoxins by hydrolysis)^21^. Afterwards, a direct method for quantification of glucuronides such as DON-3-glucuronide and DON-15-glucuronide was developed using in-house synthesized mycotoxin-standards. These analytical developments permitted the scientific community to find strong correlations between the sum of urinary DON and its glucuronides^22,23^. These investigations revealed the power of biomarker-driven research, when compared to traditional exposure assessments by combining food consumption and mycotoxin concentration data. However, previous urinary biomarker-analysis of DON represents some uncertainties and limitations: (1) biomonitoring data are dependent on the sample collection time^24^, (2) there is a lack of information on the absorption and excretion rate of DON, (3) the contribution of DON modified forms like DON-3-glucoside or acetyl-deoxynivalenol (ADON) remains unclear, and (4) left-censored data analysis remains a challenge. In addition, EFSA recommends inter-laboratory validation and standardisation of analytical methodologies to assess the exposure in urine. Certified reference materials should be made available, and proficiency tests should be facilitated^7^.

Clearly, there is a need to fully validate the DON toxicokinetics and its renal excretion in humans. Due to the lack of information on the DON absorption and excretion profile, the present study aimed to fully understand this complex matter. A human intervention design was set up with 20 subjects (55% women and 45% men), and an oral DON/DON-3-glucoside single dose administration at group-TDI level (1 µg/kg body weight) was monitored during a 24-h urine collection with subsequent biomarker analysis.

## Results

### Few urinary traces of DON and DON-3-glucoside after cereal-free diet

Before administration of the DON or DON-3-glucoside bolus, the volunteers collected a morning urine sample. These samples were considered as blank, and were taken into account for eventual recovery corrections after the intervention study. Table 1 pinpoints that the urine of three volunteers contained average trace levels of DON (2.18 ± 1.02 nmol, 1.77 ± 1.13 ng/mL), DON-3-glucuronide (4.76 ± 5.79 nmol, 6.42 ± 5.26 ng/mL) and DON-15-glucuronide (6.57 ± 1.60 nmol, 7.58 ± 0.65 ng/mL). DOM-1, DON-3-glucoside, 3-ADON and 15-ADON were below limit of quantification (LOQ) levels. The observed results were a good starting point to perform the intervention study, clearly indicating that possible found DON-levels originated from the DON/DON-3-glucoside bolus.

**Table 1:** Positive samples (%), average amount ± standard deviation (nmol) and maximum amount (nmol) of analysed DON forms (free DON, DON-3-glucuronide, DON-15-glucuronide, DOM-1, 3-ADON and 15-ADON) in the urine collected before the administration of the DON or DON-3-glucoside bolus (blank urine).

	No. of positive sample (%)	Average ± sd*	Maximum
DON	3 (7.5)	2.18 ± 1.02	2.93
DON-3-glucuronide	3 (7.5)	4.76 ± 5.79	11.37
DON-15-glucuronide	3 (7.5)	6.57 ± 1.60	7.56
DOM-1	0 (0)	n.a.	n.a.
DON-3-glucoside	0 (0)	n.a.	n.a.
3-ADON + 15-ADON	0 (0)	n.a.	n.a.

*Sd = standard deviation

DON = deoxynivalenol, DON-3-glucuronide = deoxynivalenol-3-glucurondie, DON-15-glucuronide = deoxynivalenol-15-glucuronide, DOM-1 = deepoxy deoxynivalenol, DON-3-glucoside = deoxynivalenol-3-glucoside 3-ADON = 3-acetyldeoxynivalenol and 15-ADON = 15-acetyldeoxynivalenol.

N.a. = not applicable.

### DON and DON-3-glucoside are rapidly excreted within 24 hours

**Figure 1:**
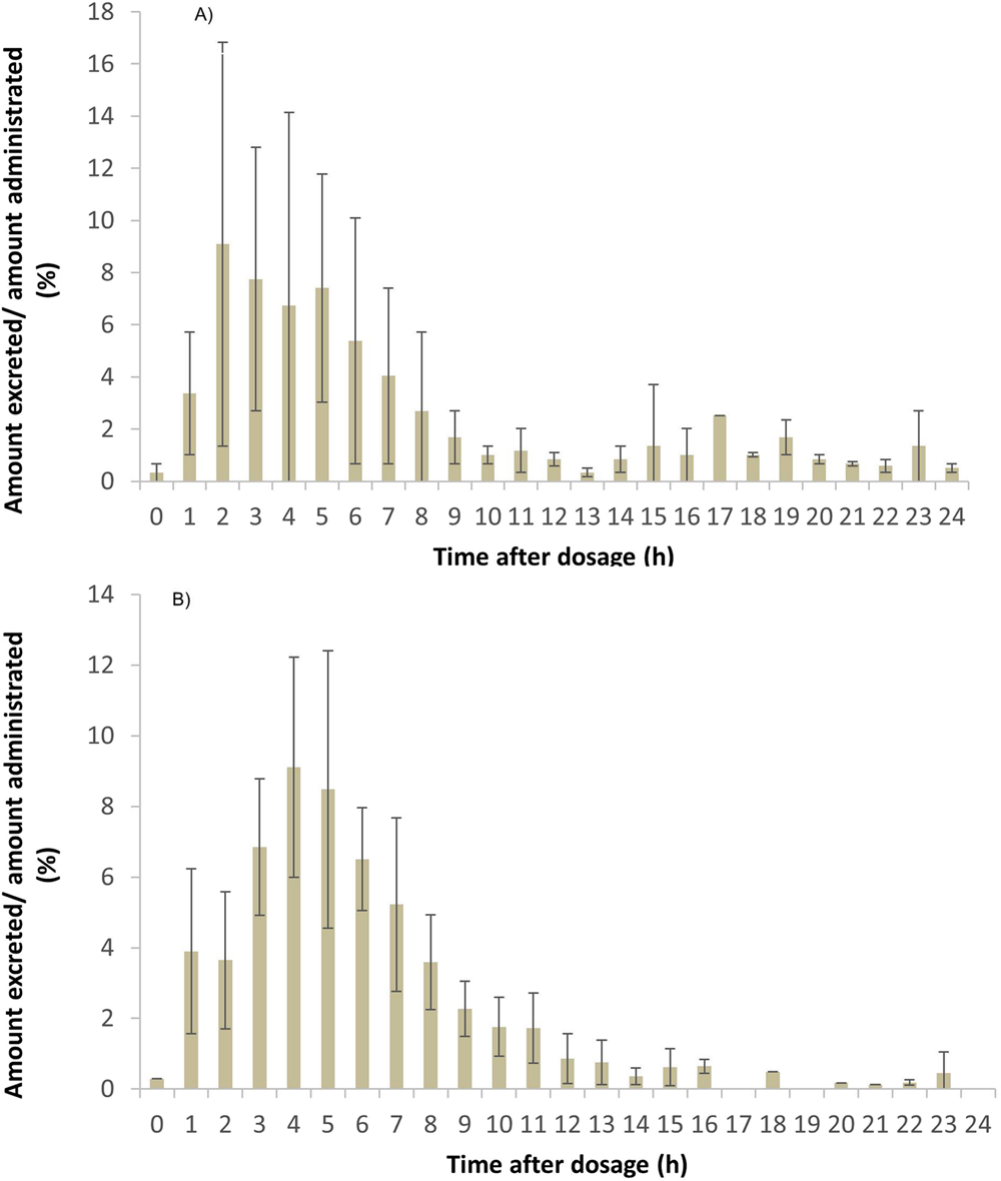
Ratio of the total DON amount excreted in urine to the total amount of administered deoxynivalenol (**A**) and deoxynivalenol-3-glucoside (**B**), during the 24 hours of urine collection. The hourly urine collection intervals were assembled based on the various irregular time points of voided urine (see Materials and Methods section).

**Figure 2:**
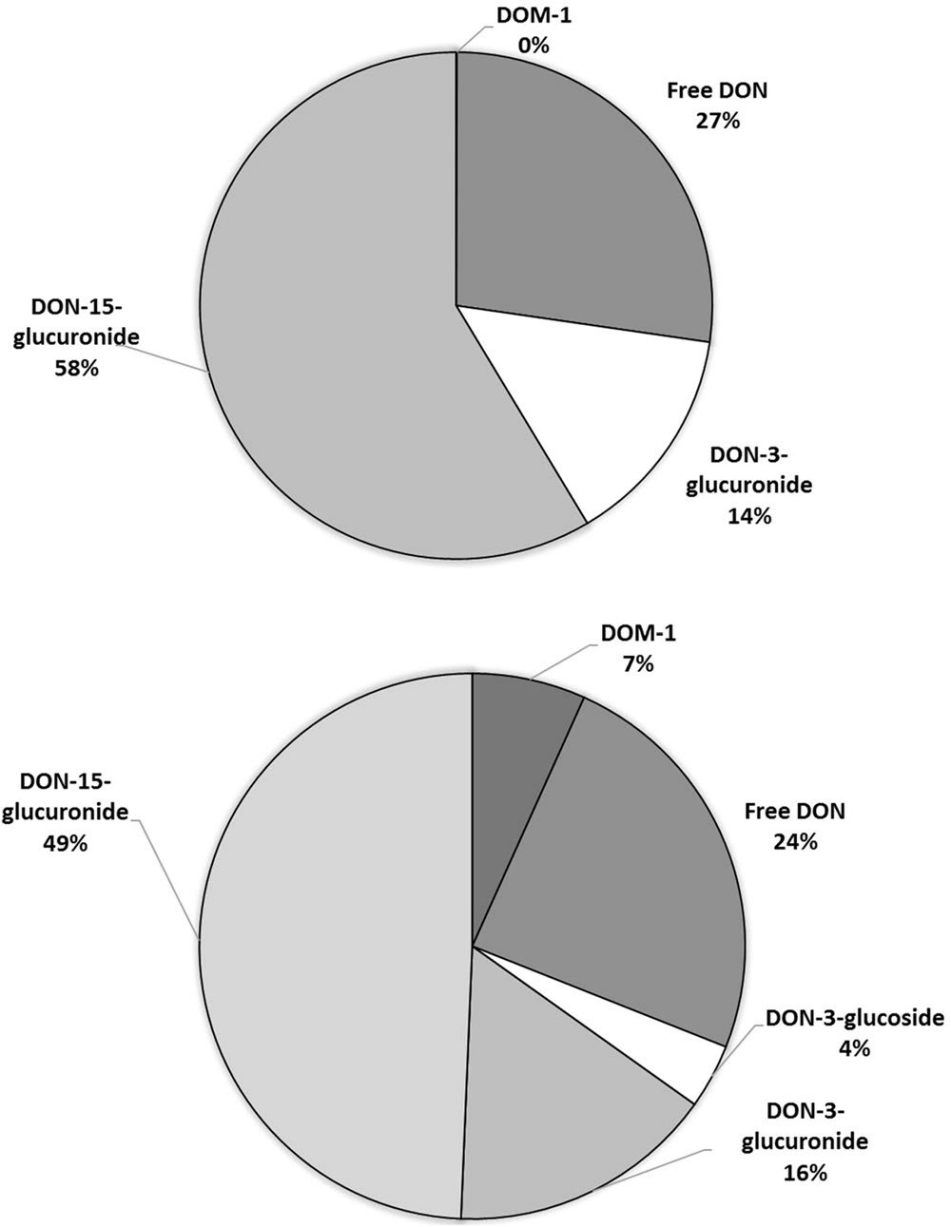
Mycotoxins recovered in urine during 24 hours after deoxynivalenol (**A**) and deoxynivalenol-3-glucoside (**B**) administration. DON = deoxynivalenol, DON-3-glucuronide = deoxynivalenol-3-glucuronide, DON-15-glucuronide = deoxynivalenol-15-glucuronide and DON-3-glucoside = deoxynivalenol-3-glucoside. DOM-1 = deepoxy-deoxynivalenol.

The urinary biomarker analyses revealed that DON was rapidly excreted within 24 hours, as the recovered total DON (free DON + DON-glucuronides) was 64.0 ± 22.8%. Moreover, a large amount of the total DON was excreted in the first hours (<6 h) after DON administration, and gradually reduced in the following hours (≥6 h) (Fig. 1). 3-ADON, 15-ADON and DON-3-glucoside were not detected. This last finding confirms that the human body is not able to metabolize the orally administered DON to acetylated DON or DON-3-glucoside. The quantified part of total DON constituted of 58.2 ± 8.74% DON-15-glucuronide, 14.4 ± 6.72% DON-3-glucuronide and 27.4 ± 11.8% free DON (Fig. 2). An average of approximately 72.6 ± 11.8% of total urinary DON was in its glucuronidated state. DOM-1 was only found in 2 volunteers, and in small amounts close to the limit of detection (LOD) (0.05 ± 0.17% of the administered dose).

DON-3-glucoside was also absorbed, distributed, metabolized and excreted rather rapidly with a total recovered DON fraction of 58.2 ± 16.0% within 24 hours. However, compared to the total DON excretion the recovery was slightly delayed. The largest amounts of excreted total DON were observed 5 to 7 hours after oral exposure (Fig. 1). The quantified part of total DON constituted of 49.1 ± 5.7% DON-15-glucuronide, 15.7 ± 4.2% DON-3-glucuronide, 3.7 ± 3.6% DON-3-glucoside, 7.0 ± 5.8% DOM-1 and 24.3 ± 5.2% free DON (Fig. 2).

### DON-15-glucuronide is the main urinary DON-biomarker

The abundant presence of DON-3/15-glucuronides confirms their validation as suitable DON and DON-3-glucoside biomarkers. Although there was not a correlation between DON and conjugated DON forms, an overall correlation of 0.61 between DON-3-glucuronide and DON-15-glucuronide was obtained for the 24 hours. Additionally, DON-15-glucuronide is the major urinary biomarker after DON administration, with a constant ratio around 4/1 (DON-15-glucuronide/DON-3-glucuronide). DON-3-glucoside has a similar excretion profile with DON-15-glucuronide being the most abundant metabolite.

### Inter-individual differences appear among sexes

**Figure 3:**
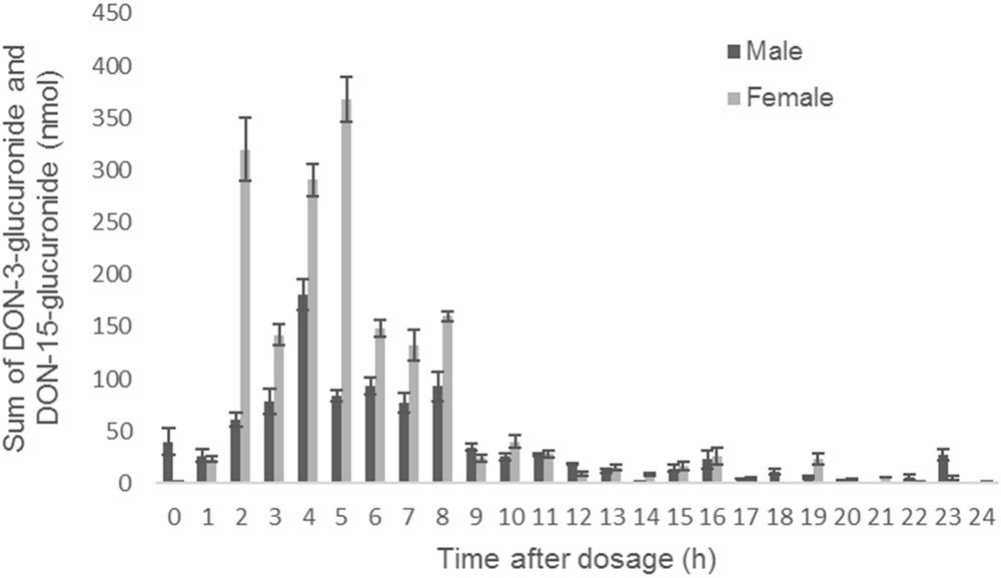
Average sum and standard deviation of deoxynivalenol-3-glucuronide and deoxynivalenol-15-glucuronide (nmol) recovered in urine during 24 hours after mycotoxin administration. DON-3-glucuronide = deoxynivalenol-3-glucuronide, DON-15-glucuronide = deoxynivalenol-15-glucuronide.

When comparing the biomarker results for total DON among sexes, a significant difference was observed after the 24-hour collection (p = 0.0032): women excreted more total DON than men. Higher concentrations of DON-15-glucuronide (p = 0.0140) and DON-3-glucuronide (p = 0.0087) were recovered in the samples originating from the female volunteers after DON and DON-3-glucoside administration (Fig. 3). After one single day 44.6 ± 13.9% (men) and 72.7 ± 13.5% (women) was recovered as total DON of the administered bolus. Men excreted 25.9 ± 9.9% of the administered dose as DON-15-glucuronide within 24 hours, while women excreted 41.7 ± 11.2%. DON-3-glucuronide was excreted at different levels: 5.6 ± 2.0% (men) and 12.6 ± 3.9% (women). A significant difference among the total glucuronides was obtained among the sexes (p = 0.0190), wherein an average of 57.2 ± 12.2% was observed for women and 31.5 ± 10.0% for men (Fig. 3).

## Discussion

The design of applying a three-day cereal-free diet proved to be sufficient. A similar study with one volunteer used a two-day cereal-free diet-design^23^, where no DON or other DON-metabolites were detected in the morning blank urine. Nagl *et al*. (2010)^14^ observed few DON traces in pig urine after a three days DON-free diet, and Vidal *et al*.^8^ detected negligible amounts of DON and metabolites in the urine of 23 volunteers after 5 days. We only detected the mycotoxins in three participants and the concentrations were so low that therefore we considered as negligible. The traces of DON found in the blank morning urines could be caused by diet unconscious mistakes made by volunteers, or other sources of probable DON-exposure (*i.e*. air or dust)^25^.

Similar recovery percentages have been detected in other studies: Warth *et al*.^23^ observed a recovery of 68% after DON administration, however, the value was obtained from only one volunteer after a 24-hours urine collection. A similar recovery (72.3%) of DON was determined based on the first-morning void urine samples in adults from the United Kingdom^26^. Collectively based on these studies, this recovery percentage can now be used in DON exposure-estimation studies, however, it is important to notice that the bolus, containing DON or DON-3-glucoside, was an aqueous solution administered at an empty stomach. In this manner, DON is likely more readily available for absorption, than a dose immerged into foods^27^.

So, a large part of DON was rapidly absorbed and excreted as glucuronides via the urine. Some animal studies proved that besides a fast urinary clearance, DON was also detectable in plasma immediately after ingestion (<30 min), but rapidly cleared from the blood indicating a fast absorption and distribution^7,12,28–30^. The fast urinary DON excretion is in accordance with a study, where most of the DON was excreted within 3 hours after intravenous dosing in pigs, while a minor fraction of the administered toxin was slowly excreted in the following hours (>3 hours)^27^.

In our study, free DON represented approximately 25% of the total urinary DON. Previous results in humans showed a similar proportion of free DON in urine. In Italy, an average of 30% of DON in urine was present^21^, while in the United Kingdom^26^ and in Spain^8^ a value of 20% was observed. The remaining portion was present in the glucuronidated form. These data suggest that the majority of DON was converted to glucuronides at some point from ingestion to urinary excretion.

The sum of DON-3-glucuronide and DON-15-glucuronide was similar as previously reported levels: 76% (range 72–80%)^23^, 66–90%^7^, 91% (range 85–98%)^26^, 86% (range 79–95%)^31^, and 92%^9^. Moreover, in recent studies, DON-15-glucuronide was identified and validated as the major DON biomarker in human urine as we observed in our results^9,20^. About 79.1 ± 18.01% of total glucuronides is derived from this metabolite, while DON-3-glucuronide accounted for approximately 20.9 ± 18.01%. The derived ratio of DON-15-glucuronide/DON-3-glucuronide is 4/1. These results seem to be consistent with another study that observed approximately 75% (DON-15-glucuronide) and 25% (DON-3-glucuronide) of total glucuronides in Austrian adults’ urine^32^.

The DON-bolus was mainly excreted during the first hours of urine collection (Fig. 1), and differences among the first 4 hours, 8 hours and 24 hours were detected (p = 0.0093). This means that the total DON amount in the urine was reduced over time. The significant difference between the first 4 hours and 8 hours was caused by free DON (p = 0.0018), as no discrepancies were observed for the glucuronides between these intervals. On the contrary, a subsequent significant difference between 8 hours and 24 hours after oral administration was obtained due to the high amounts of observed DON-15-glucuronide. By means of kinetic modelling (work in progress) the time to reach the maximum amount of total DON (Tmax) excreted in urine can be calculated and Tmax after the oral administration of DON or DON-3-glucoside can be compared.

**Figure 4:**
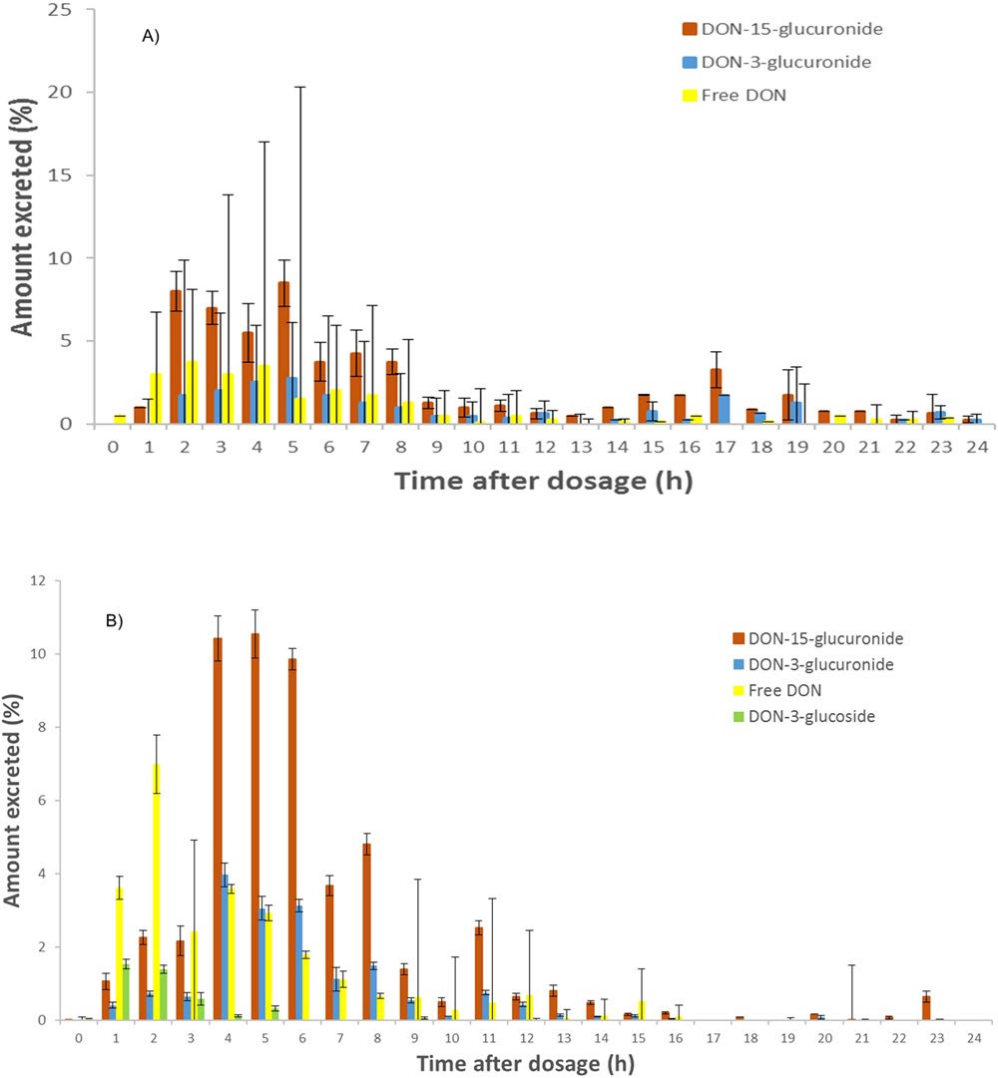
Percentage of the deoxynivalenol-15-glucuronide (DON-15-glucuronide), deoxynivalenol-3-glucuronide (DON-3-glucuronide), free deoxynivalenol (DON) and deoxynivalenol-3-glucoside (DON-3-glucoside) recovered in urine during 24 hours after DON (**A**) and DON-3-glucoside (**B**) administration.

The percentage of excreted DON forms changed over the 24 hours. Free DON was immediately detected in the urine after administration, and the amount of free DON was higher than the glucuronides in the first urinary sampling points (<2 hours) (p = 0.0018) (Fig. 4). The amount of free DON decreased over time in conjunction with an increase of the glucuronides. The DON-glucuronides (DON-3-glucuronide and DON-15-glucuronide) appeared at a later time points in the voided urine (p = 0.0003). Glucuronidation occurs in the liver, and phase II-metabolism reactions produce more polar metabolites (log D of DON-glucuronides, −5.75 at pH 7). Glucuronides are therefore easier excreted from the body in comparison to the free toxin (log D of DON, −0.97 at pH 7) 34, however the large DON and DON-3-glucoside intake caused the faster detection of DON and DON-3-glucoside in the first hours. So, DON-3-glucoside and free DON were the predominant DON compounds detected at the first 4 hours (p = 0.0024) (Fig. 4). After 4 hours of DON-3-glucoside intake, DON-glucuronides (DON-15-glucuronide and DON-3-glucuronide) appeared as the predominant DON compounds in the urine.

The transformation of DON to DON-glucuronides occurs in the liver with the aid of uridine 5′-diphospho-glucuronosyltransferase (UGT) enzymes. However, to date there is a lack of knowledge regarding the influence of UGT enzymes on DON. More than 20 UGT enzymes have been characterized, whereof 12 are commercially available. This enables an UGT-reaction phenotyping and thus the identification of potential biomarkers arising from environmental toxins. Only 2 of the 12 tested human recombinant UGTs in rat liver microsomes led to the formation of DON-glucuronides with different region specificity. UGT2B4 predominantly catalysed the formation of DON-15-glucuronide, while UGT2B7 prevailed the DON-3-glucuronide. The obtained *in vitro* data pointed out that none of the animal models were suitable for estimating the human DON metabolism with respect to the metabolite pattern and formation rate^33^. Whether the different urinary levels of DON-3-glucuronide and DON-15-glucuronide observed in our study could be resulting from a different subtype of UGT remains uncertain.

The 64% and 58% of the orally recovered dose means that the (average) absorbed DON fraction (F_abs_) was probably higher than 0.64 and 0.58. It is important to bear in mind that the absorbed fraction could be slightly higher, because some (unknown) DON metabolites or DOM-1 could be present in the analysed urine. However, as stated in the results section, the DOM-1 levels were below the LOD. On the other hand, other DON conjugates can be present in the urine^34^. Previous studies identified these conjugates as DON glucuronides (deoxynivalenol-7-glucuronide), DON sulphates (deoxynivalenol-3-sulphate and deoxynivalenol-15-sulphate) and acetyl DON sulphates (3-acetyldeoxynivalenol-15-sulphate and 15-acetyldeoxynivalenol-3-sulphate)^35^. In the current study, these metabolites were not included because 1) no reference standards are available to quantify these metabolites, and 2) low concentration levels observed in the analysed urines. Pestka *et al*. (2017) showed that DON sulphates (deoxynivalenol-3-sulphate and deoxynivalenol-15-sulphate) represented less than 1.5% of the orally administered DON in mice within 24 hours^36^. So, the total DON in urine could presumably be slightly higher based on these facts. However, it should be noted that the recovery of total DON after single dose administration of DON or DON-3-glucoside varied from 0.30 to 0.98 and 0.26 to 0.84, respectively, if 100% admission is assumed after oral administration. Particularly after DON administration, total DON recovery was more than 90% in three volunteers. This indicates that also inter-individual differences in absorption, distribution, metabolism and excretion (ADME) appeared.

Prelusky *et al*. (1986) pointed out that between 54–75% of an oral dose of DON administered to sheep was recovered in the faeces as DON and DOM-1^37^. Sheep belong to the poly-gastric animal species, and their gastro-intestinal bacterial activity is able to convert DON into DOM-1, resulting in the increase of DON forms in the faeces instead of the urine. This specific feature enables ruminants to massively lower the amount of circulating free DON and consequently these animal species are almost insensitive to acute oral intoxication of DON^38^.

However DOM-1 is a minor metabolic transformation product of DON in humans^7^. Due to the high absorption of DON by the small intestine, bacterial transformation of DON in DOM-1 can only be possible when reaching the colon. This explains why only a low percentage of ingested DON is found in the faeces of mono-gastric animals (humans, pigs, rodents)^14,27,38^. Moreover, humans may lack the relevant microflora for inducing the conversion to DOM-1. Therefore, DOM-1 is not suggested as a reliable urinary biomarker for DON exposure^7,26,39,40^. The collection of faeces from volunteers could permit to elucidate the percentage of DON excreted in faeces, however faecal material was not collected.

Besides the excretion in urine and faeces, a small amount can be recovered in the bile, as observed in a study on sheep after oral exposure. The biliary excretion does not play any important role in the elimination of DON from sheep, however, the low recovery observed in sheep could be due to the subsequent conversion of DON to unidentified metabolites in the liver^37^.

3-ADON and 15-ADON are rapidly metabolized to free DON when ingested^12^. Several reports have pointed out that acetylated DON forms are common in cereals and cereal-based products^41^, however, urinary analysis did not or scarcely reveal these forms^8^. This phenomenon confirms the fast transformation of acetylated DON to free or glucuronidated DON. The authors agree with the consideration of JECFA and EFSA to include ADON and DON-3-glucoside into the total dietary DON exposure^7^.

The average recovery percentage of DON-3-glucoside (58%) was slightly lower than DON (64%) but the differences were not statistical significant (p = 0.1951). The lower DON-3-glucoside excretion was also previously observed in humans^23^ and pigs^14^. However, DON-3-glucoside absorptions found in pigs were 16^12^ and 40%^14^, so the pigs model can be questioned to be compared with human. The different recovery is probably caused by the lower bioavailability of DON-3-glucoside, and is in accordance with the lower absorption of DON-3-glucoside by human intestinal Caco-2 cells^42^. It is questionable whether this slight difference in (apparent) bioavailability of DON and DON-3-glucoside should be taken into consideration in risk assessment studies. The total amount of DON and DON-glucuronides observed in urine after oral administration of DON-3-glucoside was approximately 51% indicating that more than half of the DON-3-glucoside dose was converted to DON before being metabolised to DON-glucuronides and excreted (Fig. 2).

Almost all DON-3-glucoside was, compared to DON, relatively rapidly excreted through the urine so, it means that it was probably also relatively rapidly absorbed, distributed and metabolized. The fast ADME process was also confirmed after an intravenous administration of DON-3-glucoside in pigs^14^. However, this kinetic process was slower than for DON. While after DON administration the major part of total DON was excreted in the first 4 hours, for DON-3-glucoside this was achieved in the first 8 hours. This delay is probably caused by the cleavage of DON-3-glucoside to DON during digestion^14^, as it also have been demonstrated for phenolic compounds^43^. The transformation of DON-3-glucoside to DON seems common in animals as similar observations were made in rats and in pigs^14^. However, the slightly slower ADME process of DON-3-glucoside, as compared to DON, does not imply that cleavage of DON-3-glucoside to DON occurs in the large intestine as has been suggested by other investigators.

It is worth to mention that only a small proportion of the total DON-3-glucoside (58.8 ± 16.0%) originated from DON-3-glucoside (3.70 ± 3.64%). The remaining part was excreted as DON-15-glucuronide (49.14 ± 5.74%), DON-3-glucuronide (15.74 ± 4.15%), free DON (24.33 ± 5.23%) and DOM-1 (7.01 ± 5.81%) (Fig. 2). The low quantity of DON-3-glucoside in the urine supports the theory that DON-3-glucoside is transformed to DON upon digestion. Consequently DON-glucuronides and DON have a similar occurrence pattern as observed in the DON administration. On the other hand, the presence of DOM-1 was higher than after the DON administration. DON-3-glucoside is probably metabolized to DOM-1 in the gut, and later absorbed and excreted through the urine. The transformation of DON-3-glucoside to DOM-1 is supported by the large presence of DOM-1 found in faeces after DON-3-glucoside administration in rats^44^.

DON-3-glucoside was excreted mainly as DON-3-glucoside during the first hours after oral administration (Fig. 4). Thus, different total DON amounts were detected during the three studied periods (p = 0.0094). Overall, the total DON amount in the urine was mostly cleared after 12 hours. DON and DON-glucuronides were scarcely detected in the first period, but after 4 hours an increase was observed.

Data from human cohort studies have shown ambiguous differences among males and females. Turner *et al*.^26^ found no significant difference in the urinary profile of males and females (n = 35)^26^. These results were in agreement with those obtained by Rodriguez-Carrasco *et al*.^45^ (n = 54) and Solfrizzo *et al*.^21^ (n = 52). Results obtained from Ediage *et al*.^46^ (n = 160) are in line with the current study, where females had significantly higher urinary DON-concentrations than the opposite sexe. However, the described studies were conducted on limited sample numbers that are unlikely to represent a large population. Therefore, the evidence to support this phenomenon in men and women are difficult^39^. To get the full picture of the gender influence on the excretion profile, additional studies will be needed with larger sample sizes. A possible explanation is that women have a higher UGT-enzyme expression, and men have a probable larger organ and plasma volume^21^. But, it remains vague which gender in humans expresses more UGT enzymes^39^. A rodent trial on DON exposure demonstrated that female animals had a higher enzyme expression than males, which suggested the gender difference in the response to DON^47^. Another possible metabolic difference is that males have a reduced capacity of glucuronidation. Sex differences have previously been identified at mRNA levels of UGT enzymes which are responsible for DON glucuronidation^47^. Overall, these data suggest that males may differ from females in absorption and/or clearance of DON.

The consumption of coffee could also affect DON excretion. Contrary to what was expected, coffee consumers excreted less free DON (6.12 ± 3.05%) during the first 4 hours after DON administration than the non-consumers (14.9 ± 5.46%) (p = 0.0269). Caffeine stimulates diuresis^48^, and therefore more DON excretion was expected. The limited number of volunteers could affect the results (n = 6, indicators of coffee consumption), and further studies are necessary to draw a more reliable conclusion on the effect and mechanism of coffee on the human DON toxicokinetics.

Other factors (body mass index, age and smoking) were investigated, but did not show any significant difference in the DON excretion. Age has been widely studied in DON exposure studies, and showed in some cases an influence in the total urinary DON. A study performed in Italy, Norway and the UK (2015) showed that age significantly affected the urinary DON concentration^49^. They observed a 2.3 to 2.6 fold higher total DON concentration in children compared to adults. The current study did not include children for ethical reasons. In our study no differences were observed in the range of 18 to 61 years. The larger DON concentration in children could be caused by the immature liver function (in comparison with adults), resulting in a lower expression of the UGT-enzyme^39^.

The body mass index did not influence the recovered DON-biomarkers in urine, which was also confirmed in other studies^26^. DON is a hydro-soluble molecule, and thus hardly absorbed in the lipid parts of the body. The percentages that were compared among the different groups were obtained by dividing the excreted amount with the administered amount (both in nmol), whereby the administered amount (in µg) was already corrected for body weight (1 µg/kg body weight).

Regarding smoking, there were only two subjects among the volunteers. Their DON excretion did not show differences compared to non-smokers. Smoking can cause inter-individual variabilities in the excretion profile of residues and contaminants, mainly because its relation with a decreased health-state which could affect ADME^50^. A higher number of smoking subjects should be taken in future research to thoroughly study the possible effect of smoking on DON excretion.

In conclusion, this study shows that the urinary analysis of DOM-1, DON-3-glucoside and/or ADONs to evaluate DON-exposure is not relevant. Fortunately, most exposure studies do already focus on the three most abundant biomarkers, namely DON, DON-3-glucuronide and DON-15-glucuronide. Moreover, the current results show that the use of morning urine is not adequate to assess DON exposure. Most likely, only DON intake during the evening meal is somehow reflected in the morning urine assuming that no urine is voided before bedtime. The authors therefore suggest to collect at least 16-hours urine to have a representative view on DON-consumption. Additionally, this study shows that a preferred urinary biomarker of exposure to DON, DON-3-glucoside or a mixture of both is DON-15-glucuronide. Kinetic modelling of the urinary excretion data (work in progress) will reveal whether DON-15-glucuronide alone, both glucuronides together or a combination of DON and its two glucuronides is the preferred (set of) biomarker(s). For now, using the total amount of DON and its two glucuronides excreted in 24 hours (on a molar basis), a range of recovery of 58 to 64% can be used to cover for the exposure to either DON-3-glucoside, or DON, or a mixture of both. Finally, the DON excretion depends on the gender, and women excretes more DON than women.

## Materials and Methods

### Chemicals and preparation of the DON/DON-3-glucoside bolus

The individual mycotoxin solid calibration standards (1 mg) of DON, 3-ADON, 15-ADON, DOM-1, DON-3-glucoside and isotope-labelled (^13^C_15_) DON (internal standard) were obtained from Sigma Aldrich (Bornem, Belgium). All mycotoxin solid standards were dissolved in methanol (1 mg/mL) and were storable for a minimum of 1 year at −18 °C^51^. The working solutions of DON, 3-ADON, 15-ADON, DOM-1, DON-3-glucoside and isotope-labelled (^13^C_15_) DON were prepared in methanol, and stored at −18 °C. DON-3-glucuronide was supplied by Dr. Huybrechts (CODA-CERVA, Tervuren, Belgium). Water was obtained from a Milli-Q^®^ SP Reagent water system from Millipore Corp. (Brussels, Belgium). Disinfectol^®^ (denaturated ethanol with 5% ether) was supplied by Chem-Lab (Zedelgem, Belgium). Methanol (LC-MS grade) was purchased from BioSolve (Valkenswaard, the Netherlands), while acetonitrile (Analar Normapur) was obtained from VWR International (Zaventem, Belgium). Acetic acid (glacial, 100%) and formic acid (98–100%) were supplied by Merck (Darmstadt, Germany). Magnesium sulphate anhydrous (>99.5%) was supplied by Alfa Aesar (Haverhill, Massachusetts, US). Sodium chloride (>99.5%) was supplied by VWR Chemicals (Radnor, Pennsylvania, US). The DON-bolus and DON-3-glucoside-bolus were prepared by dissolving 5 mg of mycotoxin (DON or DON-3-glucoside) in 0.5 L of drinkable water. After dissolution, the exact concentrations (n = 5) were verified trough LC-MS/MS. Individual aliquots, based on the individual weight of each subject and the group-TDI of DON or DON-3-glucoside, were made using this solution.

### Study design of the human intervention study

The human intervention study was conducted according to the guidelines laid down in the declaration of Helsinki, and was approved by the Ethical Committee of the Ghent University Hospital (B670201630414). Participants, members of the research group and familiars, were contacted providing an invitation letter and can register for the study within two weeks after the call. All participants signed an informed consent. Each participant was informed on his/her right to withdraw from the study at any time and to consult a doctor immediately and inform us if they felt not right during the study but no adverse event were reported for any volunteers. Besides, no medical examinations or interventions were carried out in this study. The study was performed with 20 volunteers throughout an intervention and longitudinal trail, and recruited in Flanders (Belgium). The subjects included 20 healthy adults with 11 women (55%) and 9 men (45%) (mean age 32 years, range 18–61 years). The volunteers were instructed to file a socio-demographic questionnaire involving details on age, gender, length, body weight, smoking, diseases, drugs or supplements, pregnancy, breast-feeding, diet and daily coffee consumption. The following persons were excluded from the study: (1) pregnant or breast-feeding women due to the potential risk to both mothers and foetuses; (2) persons with severe problems with liver, bile or kidney due to related risks for interferences with the mycotoxin metabolism.

**Figure 5:**
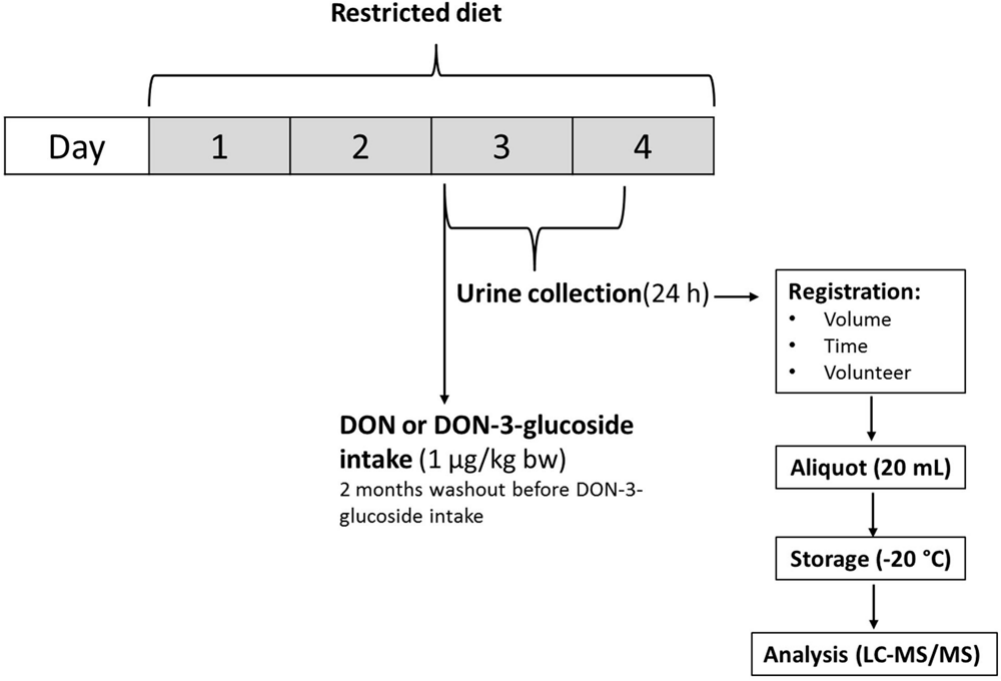
Scheme of the study design.

The subjects followed a strict cereal-free diet during 3 days (Fig. 5). Cereal-based foods and foods containing possible traces of wheat, rye, oats, maize and rice were restricted (*i.e*. bread and bread-based products, breakfast cereals, oat meal, muesli, cereal bars, waffles, cakes, (un)pealed rice, polenta, pizza, tortilla, pasta, popcorn, cereal-based chips, maize, beer, wine, pan meal and sauce-binders). The subjects were asked to detail their daily food intake through a questionnaire. On the third day in the morning, the subjects received an oral bolus of DON or DON-3-glucoside based on the TDI (1 µg/kg body weight) and their body weight. The volunteers did the study twice: 1) DON administration and 2) DON-3-glucoside administration, between the two mycotoxin administration there was a wash-out period of two months where volunteers did not have to follow any special requirements on diet. In addition, a control group of 4 volunteers followed the same protocol, however did not receive a bolus of DON or DON-3-glucoside after the cereal-free diet.

### Collection of the 24-hours urine

A blank urine sample was collected before the administration of the DON/DON-3-glucoside bolus at day 3. From the moment of administration, the 24-hours urine sample collection was requested. For every sampling point, time (hour of urine collection) and voided volume was recorded. Each participant had an average of 8 ± 2 sampling points. All samples were individually aliquotted to 20 mL, and stored in the freezer at −20 °C upon analysis. These aliquots were subdivided into two parts where one was used for biomarker-analysis and one as back-up sample.

### Sample preparation and targeted LC-MS/MS analysis

To 18 mL of acetonitrile/water/formic acid (52/45/3, v/v), 2 mL of the thawed urine sample was added in combination with 4 g of anhydrous magnesium sulphate and 1 g of sodium chloride. After addition, the sample was vigorously shaken by hand. Then, the samples were placed on an Agitator decanter overhead shaker for 30 minutes (Agitelec, J. Toulemonde & Cie., Paris, France), and centrifuged at 4,000 *g* for 6 minutes. Seven mL of the non-polar fraction was evaporated to dryness under a gentle nitrogen air at 40 °C. Finally, the residue was redissolved in 250 µL injection solvent (methanol/water, 10/90, v/v).

A Waters Acquity UPLC system coupled to a Quattro XEVO TQS mass spectrometer (Waters, Manchester, UK) was used to analyse the urine samples. Data acquisition and processing was performed with MassLynx™ version 4.1 and QuanLynx^®^ version 4.1 software (Waters, Manchester, UK). A Waters Acquity UPLC^®^ HSS T3 (2.1 × 100 mm, 1.8 µm) column was applied (Waters, Manchester, UK). Two different mobile phases were used, and consisted of water/acetic acid (99.9/0.1, v/v (A)) and methanol/acetic acid (99.9/0.1, v/v (B)). The gradient elution program started at 99% mobile phase A. After an isocratic phase for 0.5 minutes at initial conditions, mobile phase B increased to 45% in 6 minutes. Then, a plateau phase for 1.5 minutes was enhanced with 99% mobile phase B. An equilibration step of 1.5 minutes was introduced, resulting in a total run time of 9 minutes. The flow rate was set at 0.4 mL/min. The mass spectrometer was operated in both positive and negative electrospray ionisation mode (ESI^+^/ESI^−^). The capillary voltage was 30 kV, and nitrogen was applied as spray gas. The source and desolvation temperatures were set at 150 °C and 200 °C, respectively. The argon collision gas pressure was 9 × 10^–6^ bar, the cone gas flow 50 L/h and the desolvation gas flow 500 L/h. Two selected reaction monitoring (SRM) transitions with a specific dwell-time were optimised for each analyte, in order to increase the sensitivity and the selectivity of the mass spectrometric conditions (Table 2). The developed LC-MS/MS method was successfully validated based on the European Commission Decision 2002/657/EC laying down the rules for the analytical methods to be used in the testing of official samples^52^. Matrix-matched calibration plots were constructed for the determination of the analytes. ^13^C_15_ DON was used as internal standard in the multi-mycotoxin analysis. Evaluating the linearity, the homogeneity of variance was checked before fitting the linear model. The linearity was interpreted graphically using a scatter plot. The precision was calculated in terms of the relative standard deviation (RSD). Limit of detection (LOD) was calculated as three times the standard error of the intercept, divided by the slope of the standard curve; the limit of quantification (LOQ) was similar, differing by six times the standard error. The calculated LOD and LOQ were verified by the signal-to-noise ratio (s/n), which should be more than 3 and 10, respectively according to the IUPAC guidelines^53^. The results of the performance characteristics of the LC-MS/MS method were in agreement with the criteria mentioned in European Commission Decision 2002/657/EC (Table 3).

**Table 2:** The optimized ESI-MS/MS parameters for the identification and quantification of deoxynivalenol (DON), deoxynivalenol-3-glucoside (DON-3-glucoside), deoxynivalenol-3-glucuronide/deoxynivalenol-15-glucuronide (DON-3-glucuronide/DON-15-glucuronide), 3-acetyldeoxynivalenol/15-acetyldeoxynivalenol (3-ADON/15ADON), de-epoxy-deoxynivalenol (DOM-1) and isotope-labelled (13C15) deoxynivalenol (13C15 DON) in urine.

**Mycotoxin**	Precursor ion (m/z)	Product ions^a^ (m/z)	CE^a,b^ (eV)	CV^c^ (v)	Retention time (min)
DON	297.0	249.0/231.0	9/9	40	3.99
DON-3-glucoside	459.1	168.1/132.0	10/9	15	3.90
DON-3-glucuronide/DON-15-glucuronide	471.0	113.0/193.0	30/24	60	3.65/3.78
3-ADON/15-ADON	339.0	231.0/203.1	15/9	15	5.69
DOM-1	281.1	215.1/233.1	9/9	40	4.83
^13^C_15_ DON	311.9	262.9/130.5	10/10	30	3.99

^a^Values are given as quantifier ion/qualifier ion.

^b^CE: Collision energy.

^c^CV: Cone Voltage.

**Table 3:** Validation results of the analysed mycotoxins in urine.

Mycotoxin	LOD^a^ (ng/mL)	LOQ^b^ (ng/mL)	Calibration Range (ng/mL)	R^c^ (mean)	Apparent recovery (%)	SE^d^	RSD_r_^e^ (%)	RSD_R_^f^ (%)	U^g^ (%)
DON	0.2	0.4	0.5–20	0.99	103.3	7.6	5.5	6.6	14.8
DON-3-glucoside	0.3	0.6	0.5–20	0.99	97.8	8.7	0.9	3.7	8.3
DON-3-glucuronide/DON-15-glucuronide	0.5	1.0	0.5–20	0.99	111.3	9.9	7.2	10.1	20.8
3-ADON/15-ADON	0.1	0.2	0.5–20	0.99	105.1	7.7	2.5	5.4	13.2
DOM-1	0.6	1.2	0.5–20	0.99	101.3	0.5	2.8	4.5	10.0

^a^LOD = Limit of detection.

^b^LOQ = Limit of quantification.

^c^Mean value.

^d^SE = Standard error of mean.

^e^RSDr = relative standard deviation.

^f^RSD_R_ = relative standard deviation inter-day precision.

^g^U = measurement uncertainty.

DON = deoxynivalenol, DON-3-glucoside = deoxynivalenol-3-glucoside, DON-3-glucuronide = deoxynivalenol-3-glucuronide, DON-15-glucuronide = deoxynivalenol-15-glucuronide, 3-ADON = 3-acetyldeoxynivalenol, 15-ADON = 15-acetyldeoxynivalenol, DOM-1 = de-epoxy-deoxynivalenol.

### Calculations

All obtained results were carried out on molar basis taking the molecular weight of the analytes (DON, 296 g/mole; DON-3-glucoside, 458 g/mole; DON-3-glucuronide and DON-15-glucuronide, 472 g/mole; DOM-1, 280 g/mole; 3-ADON and 15-ADON, 338 g/mole) and the total volume of each sampling point into account. Associations between independent and dependent variables were assessed by using univariate and multivariable linear regression models. Independent variables included age (years), gender, body mass index (BMI), and the consumption of coffee. The statistical analysis was performed using the software Microsoft Excel^®^ 2010 and SPSS^®^ 15.0.

The excretion profiles shown in Figs 1 and 4 are based on the values obtained each hour after the bolus administration from a varying number of volunteers per time period. For instance, results from 61 to 120 minutes are clustered in the second hour. The bars from Fig. 3 were based on the values of DON-glucuronides (DON-15-glucuronide + DON-3-glucuronide) obtained each hour after the bolus administration.

### Clinical Trial Register

The study has been registered in the Dutch Trial Register (NTR). The number assigned is NTR6902. The date registered in NTR: 08/12/2017. You can visit the link of the clinical trial register at: http://www.trialregister.nl/trialreg/admin/rctview.asp?TC=6902.

## Electronic supplementary material

Trial Protocol
