# Supplementary material for: Humans significantly metabolize and excrete the mycotoxin deoxynivalenol and its modified form deoxynivalenol-3-glucoside within 24 hours
Source: Sci Rep. 2018 Mar 27;8:5255. doi: 10.1038/s41598-018-23526-9 (PMC5869592; doi:10.1038/s41598-018-23526-9)
Supplement: Supplementary file 1 — Trial Protocol [file 41598_2018_23526_MOESM1_ESM.pdf]

Project summary

For the first time, a large-scale human intervention study was conducted to unravel the urinary excretion profile and metabolism of the mycotoxin deoxynivalenol (DON) and its modified form deoxynivalenol-3-glucoside (DON-3-glucoside). DON and DON-3-glucoside are common contaminants in cereals and cereal-based food products, and contribute significantly to the dietary intake of mycotoxins. A group of 20 healthy volunteers will be selected for this study. The subjects have to follow a strict cereal-free diet during 3 days. Cereal-based foods and foods containing possible traces of wheat, rye, oats, maize and rice are restricted (*i.e.* bread and bread-based products, breakfast cereals, oat meal, muesli, cereal bars, waffles, cakes, (un)pealed rice, polenta, pizza, tortilla, pasta, popcorn, cereal-based chips, maize, beer, wine, pan meal and sauce-binders). The subjects are asked to detail their daily food intake through a questionnaire. On the third day in the morning, the subjects receive an oral bolus of DON or DON-3-glucoside based on the TDI (1 µg/kg body weight) and their body weight. The volunteers did the study twice: 1) DON administration and 2) DON-3-glucoside administration, between the two mycotoxin administration there are a washout period of two months. In addition, a control group of 4 volunteers follow the same protocol, however do not receive a bolus of DON or DON-3-glucoside after the cereal-free diet. At day 3, volunteers have to collect a urine sample before the administration of the DON or DON-3-glucoside bolus. From the moment of administration, the 24-hours urine sample collection is requested. We deliver to each volunteer 12 containers (1L) to collect the urine.

Title: Metabolization and renal excretion of deoxynivalenol and its glucoside.

Date: from January to May 2017.

Protocol identifying number: NTR6902

Name sponsor/funder: Foodball project and Research Foundation Flanders (FWO)

Address: FWO, Rue d'Egmont 5, 1000 Bruxelles (Belgium)

Name and title of investigators who are responsible for conducting the research

Investigator: Prof. Sarah De Saeger
Address: Ottergemsesteenweg, 460. 9000 Gent (Belgium).
Phone Number: 0032 9 264 81 34

Responsibilities: planning and organization of the study.

Investigator: Dra. Marthe De Boevre
Address: Ottergemsesteenweg, 460. 9000 Gent (Belgium).
Phone Number: 0032 9 264 81 34

Responsibilities: planning and organization of the study.

Investigator: Dr. Arnau Vidal
Address: Ottergemsesteenweg, 460. 9000 Gent (Belgium).
Phone Number: 0032 9 264 81 34

Responsibilities: collection and analysis of the samples.

Name(s) and address(es) of the clinical laboratory(ies) and other medical and/or technical department(s) and/or institutions involved in the research.

Laboratory of Food Analysis, Department of Bioanalysis, Faculty of Pharmaceutical Sciences, Ghent University, Ottergemsesteenweg, 460. Ghent, Belgium.

Rationale & background information

The Rationale specifies the reasons for conducting the research in light of current knowledge. It should include a well documented statement of the need/problem that is the basis of the project, the cause of this problem and its possible solutions. It is the equivalent to the introduction in a research paper and it puts the proposal in context. It should answer the question of why and what: why the research needs to be done and what will be its relevance. The magnitude, frequency, affected geographical areas, ethnic

and gender considerations, etc of the problem should be followed by a brief description of the most relevant studies published on the subject.

Deoxynivalenol (DON), also known as vomitoxin, is a mycotoxin that acts as a potent inhibitor of protein synthesis, stimulates the pro-inflammatory response, cause ribotoxic stress, cytotoxicity and apoptosis, resulting on the impairment of multiple physiological functions, such as the intestinal barrier, growth, immune regulation or reproduction. Furthermore, this mycotoxin has been linked with animal and human gastroenteritis outbreaks due to acute exposition (Pestka, 2010). Despite its toxicity, DON is a highly common mycotoxin in cereals and cereals products ((De Boevre, et al., 2012; Marin, Ramos, Cano-Sancho, & Sanchis, 2013) Marin et al., 2013) and as a result it is one of the major mycotoxins in our diets. Thus, exposition studies showed the large exposure of human to this toxin with high percentages of population exceeding the tolerable daily intake (TDI) (Heyndrickx, et al., 2015; Vidal, Cano-Sancho, Marin, Ramos, & Sanchis, 2016).

Furthermore, free mycotoxins, like DON, might not be the only hazard for consumer's health, because the so-called modified mycotoxins are also widely common in food. Modified mycotoxins are toxins attached to more polar functional groups, such as glycosyl residues or sulfates, or to polymeric carbohydrates or protein matrices (Berthiller, Schuhmacher, Adam, & Krska, 2009; Rychlik, et al., 2014). The modified mycotoxins may have plant, fungal, mammalian and food processing origins. A major concern and potential risk for consumers is the possible hydrolysis of modified mycotoxins into their toxic free forms during mammalian digestion (Broekaert, et al., 2015; Grabley, Gareis, Bockers, & Thiem, 1992; V. Nagl, et al., 2014). Contrary to the wealth of information on the free mycotoxins, only limited data are available for mycotoxin derivatives in foods. The co-occurrence of free and modified DON forms has been documented in raw wheat, especially with focus on deoxynivalenol-3-glucoside (DON-3-glucoside), 3-acetyldeoxynivalenol (3-ADON) and 15-acetyldeoxynivalenol (15-ADON). Reported levels of DON-3-glucoside are variable, however, the concentration of DON-3-glucoside can be high and even the same as DON in processed cereals (De Boevre, et al., 2012). 3-ADON and 15-ADON have also been detected in cereals and cereals products with a lower incidence than DON-3-glucoside (De Boevre, et al., 2012). (Berthiller, et al., 2011) demonstrated that several lactic acid bacteria hydrolyse DON-3-glucoside in vitro, which has been a first step to prove the toxicological relevance of DON-3-glucoside. On the other hand, 3-ADON and 15-ADON are rapidly converted to DON during digestion (Broekaert, et al., 2015; Versilovskis, et al., 2012). Thus, due to the high presence of DON conjugates in food and the easy transformation of them to DON, the FAO/WHO Expert Committee (JEFCA) considered DON-3-glucoside to be an additional contributing factor to total dietary exposure to DON (JECFA, 2010).

To know the DON exposition, analysis of urinary levels of DON has been proposed due to its short excretion half-life. However, different studies showed that DON glucuronides, which are the main phase II metabolites of DON, are the most common DON form in urine, specially DON-

3-glucuronide and DON-15-glucuronide (Warth, et al., 2012). So, the analysis of glucuronides forms in urine is crucial for the study of trichothecenes biomarkers, because about 90 % of DON excreted via urine is conjugated with glucuronic acid. For the glucuronides determination, a preliminary approach was developed based on the breakage of deoxynivalenol-glucuronides and subsequent determination of “total deoxynivalenol” (sum of free and released mycotoxins by hydrolysis). Afterwards, a direct method for quantification of glucuronides such as deoxynivalenol-3-glucuronide and deoxynivalenol-15-glucuronide was developed. The analytical developments permitted to find strong correlations between the sum of urinary deoxynivalenol and its glucuronidated metabolites (Turner, White, et al., 2010; Warth, Sulyok, Berthiller, Schuhmacher, & Krska, 2013). These investigations revealed the power of biomarker driven work when compared to traditional exposure assessment by analyzing food stuff. However, the analysis of DON in urine presents some uncertainties and limitation to fully validate the DON excretion metabolism and renal excretion. Firstly, biomonitoring data may depend on the moment in time when the sample is collected (Clewell, Tan, Campbell, & Andersen, 2008). Furthermore, there is a lack of information in the absorption and excretion rate of it. Finally, the high presence of DON conjugates in food like DON-3-glucoside or acetyl-deoxynivalenol (ADONs) add more uncertainties for the correlation between urinary DON and DON intake.

Study goals and objectives

Goals are broad statements of what the proposal hopes to accomplish. They create a setting for the proposal. Specific objectives are statements of the research question(s). Objectives should be simple (not complex), specific (not vague), and stated in advance (not after the research is done). After statement of the primary objective, secondary objectives may be mentioned.

Due to the lack of information about DON absorption and excretion, the aims of this study are the description of the DON and metabolites excretions patterns and know the absorption and excretion rates of it. Moreover, the results can be useful to build an standardized method to estimate deoxynivalenol-intake by means of biomarkers.

Study Design

The scientific integrity of the study and the credibility of the study data depend substantially on the study design and methodology. The design of the study should include information on the type of study, the research population or the sampling frame, and who can take part (e.g. inclusion and exclusion criteria, withdrawal criteria etc.), and the expected duration of the study

(The same study can be described in several ways, and as complete a description of the study as possible should be provided. For example, a study may be described as being a basic science research, epidemiologic or social science research, it may also be described as observational or interventional; if observational, it may be either descriptive

or analytic, if analytic it could either be cross-sectional or longitudinal etc. If experimental, it may be described as a controlled or a non controlled study. The link below provides more information on how to describe a research study

The human controlled intervention study is conducted according to the guidelines laid down in the declaration of Helsinki, and was approved by the Ethical Committee of the Ghent University Hospital (B670201630414). Participants, members of the research group and familiars, are contacted providing an invitation letter and can register for the study within two weeks after the call. All participants have to sign an informed consent. Each participant is informed on his/her right to withdraw from the study at any time and to consult a doctor immediately and inform us if they fell not right during the study but no adverse event are reported for any volunteers. Besides, no medical examinations or interventions are carried out in this study. The study is performed with 20 volunteers throughout an intervention and longitudinal trail, and recruited in Flanders (Belgium). All people older than 18 years old can take part in the study, less pregnant or breast-feeding women due to the potential risk to both mother and foetuses and persons with severe problems with liver, bile or kidney due to related risks for interferences with the mycotoxin metabolism. The volunteers are instructed to file a socio-demographic questionnaire involving details on age, gender, length, body weight, smoking, diseases, drugs or supplements, pregnancy, breast-feeding, diet and daily coffee consumption.

The subjects have to follow a strict cereal-free diet during 3 days (Figure 1). Cereal-based foods and foods containing possible traces of wheat, rye, oats, maize and rice are restricted (*i.e.* bread and bread-based products, breakfast cereals, oat meal, muesli, cereal bars, waffles, cakes, (un)pealed rice, polenta, pizza, tortilla, pasta, popcorn, cereal-based chips, maize, beer, wine, pan meal and sauce-binders). The subjects are asked to detail their daily food intake through a questionnaire. On the third day in the morning, the subjects receive an oral bolus of DON or DON-3-glucoside based on the TDI (1 µg/kg body weight) and their body weight. The volunteers did the study twice: 1) DON administration and 2) DON-3-glucoside administration, between the two mycotoxin administration there are a washout period of two months. In addition, a control group of 4 volunteers follow the same protocol, however do not receive a bolus of DON or DON-3-glucoside after the cereal-free diet.

At day 3, volunteers have to collect a urine sample before the administration of the DON or DON-3-glucoside bolus (Figure 1). From the moment of administration, the 24-hours urine sample collection is requested. We deliver to each volunteer 12 containers (1L) to collect the urine. For every sampling point, time (hour of urine collection) and voided volume is recorded. All samples will be individually aliquotted to 20 mL, and stored in the freezer at -20°C upon analysis. The samples are analysed with a Waters Acquity UPLC system coupled to a Quattro XEVO TQS mass spectrometer (Waters, Manchester, UK) was used to analyse the urine samples. Data acquisition and processing is performed with MassLynx™ version 4.1 and

QuanLynx® version 4.1 software (Waters, Manchester, UK). A Waters Acquity UPLC® HSS T3 (2.1 x 100 mm, 1.8 µm) column is applied (Waters, Manchester, UK).

The results are submitted an statistical analysis. Firstly, all obtained results are carried out on molar basis taking the molecular weight of the analytes (DON, 296 g/mole; DON-3-glucoside, 458 g/mole; DON-3-glucuronide and DON-15-glucuronide, 472 g/mole; DOM-1, 280 g/mole; 3-ADON and 15-ADON, 338 g/mole) and the total volume of each sampling point into account. Associations between independent and dependent variables are assessed by using univariate and multivariable linear regression models. Independent variables included age (years), gender, body mass index (BMI), and the consumption of coffee. The statistical analysis was performed using the software Microsoft Excel® 2010 and SPSS® 15.0.

Figure 1. Scheme of the study design.

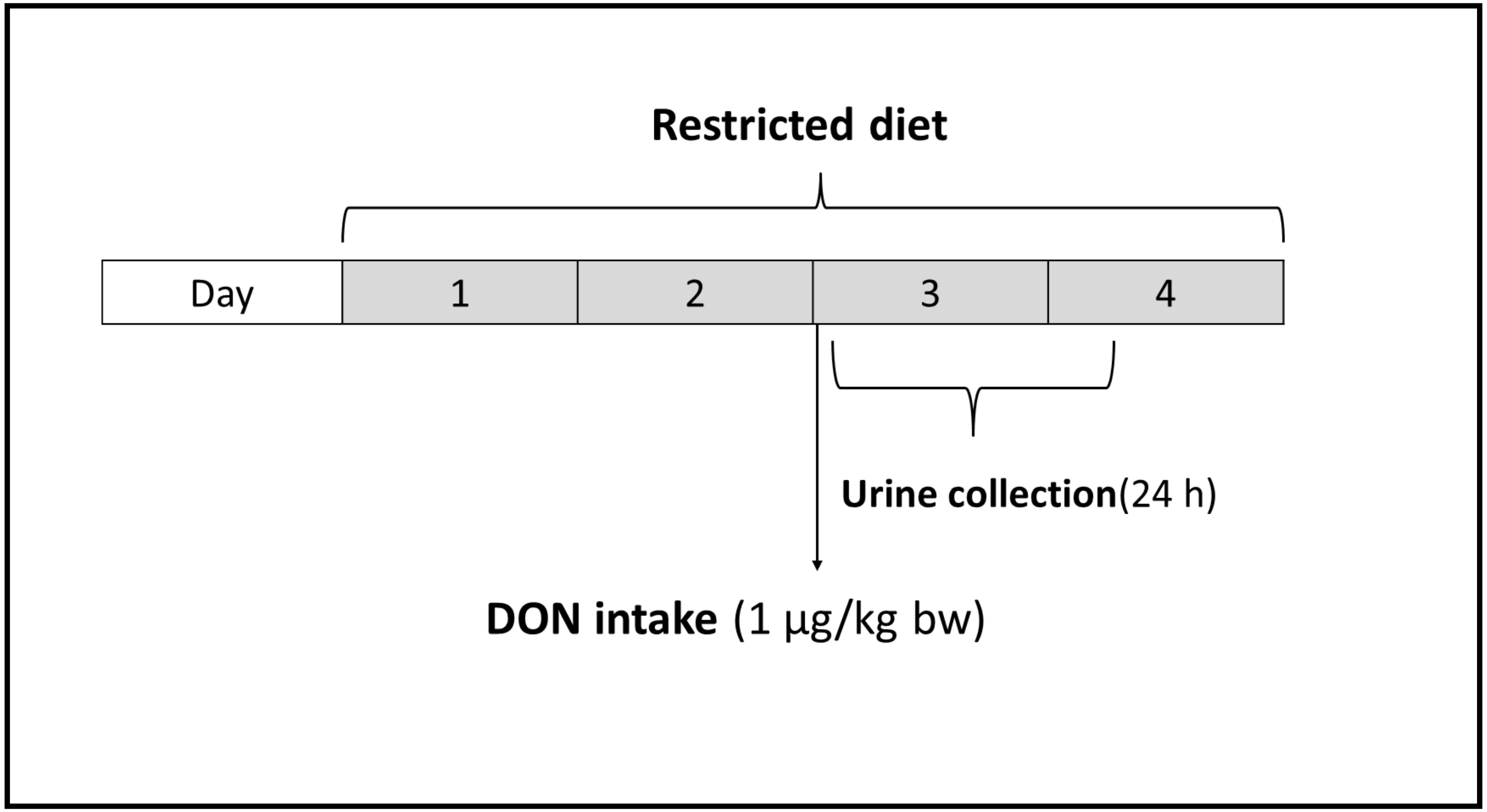

Methodology

The methodology section is the most important part of the protocol. It should include detailed information on the interventions to be made, procedures to be used, measurements to be taken, observations to be made, laboratory investigations to be done etc. If multiple sites are engaged in a specified protocol, methodology should be standardized and clearly defined.

Interventions should be described in detail, including a description of the drug/device/vaccine that is being tested. Interventions could also be in the realm of social sciences for example providing training or information to groups of individuals.

Procedures could be biomedical (collection of blood or sputum samples to develop a diagnostic test), or in the realm of social sciences (doing a questionnaire survey, carrying out a focus group discussion as part of formative research, observation of the participant's environment, etc.).

Standardized and/or documented procedures/techniques should be described and bibliographic references, if not provided earlier should be provided. Instruments which are to be used to collect information (questionnaires, FGD guides, observation recording form, case report forms etc.) must also be provided.

In the case of a randomized controlled trial additional information on the process of randomization and blinding, description of stopping rules for individuals, for part of the study or entire study, the procedures and conditions for breaking the codes etc. should also be described.

A graphic outline of the study design and procedures using a flow diagram must be provided. This should include the timing of assessments.

Volunteers have to be three days without eating cereals. It is difficult to control their diet for 24 h, so we absolutely trust in their free cereals diet. However, we know that if some volunteers do not follow the diet we will detect DON levels in the “blank” urine (the urine before toxin administration) and they have to repeat the intervention study. So, although we do not control totally the volunteers diet we have the “blank” urine as control of their good diet following. We deliver a list with the allowed and not allowed food to clarify even more the restricted food to the volunteers (Figure 2).
On the other hand, we add four volunteers which will not take toxin administration but they do not know they are not taking the toxin administration just water. The 4 control volunteers will provide us information to compare the results. The control volunteers are random selected from the 20 volunteers. 2 males and 2 females will be randomly selected for control.

The volunteers have to answer a questionnaire where we ask for factors that could affect the toxin excretion (Figure 3). Moreover, we ask again about pregnant or breast feeding people as well as

kidney, liver or bile problems to be sure again that pregnant or breast feeding women and people with kidney, liver or bile problems are not included in the study.

Stickers with the code for each sample will be delivered to the volunteers with the urine collection containers. The volunteers have to stick each code to the urine sample and they have to add the hour of urine collection for each sample. This process is explained in detail to each volunteer when they come to collect the containers, moreover, we supply a completely detailed procedure about how to take the samples, where we added a phone number contact to call in case of doubts during the study.

Once, we received the samples from each volunteer we introduced the code, the hour and the volume for each sample in excel file and we take two aliquots of 20 mL for each samples and we stored until analysis in the freezer at -20 °C.

Then, the samples are analysed by LC-MS/MS with a previously validated method.

A flow diagram with timming assessments has been made with the study design and procedures (Figure 4).

Figure 2. List with the allowed and not allowed food during the intervention diet. The list is delivered to the volunteers to be better informed about the diet to follow during the study. Moreover, we added some diet recommendations to be followed during the days of restriction diet.

Restricted food	Permitted food
• Pasta<br>• Breads or similar (small toasts, sliced bread…)<br>• Baking products (cookies, muffins…)<br>• Breakfast cereals<br>• Snacks	• Fruit<br>• Vegetable<br>• Meat<br>• Fish<br>• Nuts<br>• Dairy products<br>• Eggs
**Beverages**	
• Beer	• Other

Recommendations:

The breakfast is may be the most difficult meal during the diet because we usually bread or bakery products due to his high energy level. So, eat fruit or dairy products like yogurts is very useful to overcome the diet. Also you can add nuts to your dairy products. Nuts are very energetic and they will help you to stand until lunch time.

Eat vegetables or salads with fish, meat or omelettes can be a good option for lunch or dinner.

Figure 3. Socio-demographic questionnaire for the volunteers. The questions ask for factors that could affect the excretion rate. They have to answer all the questions.

Socio-demographic questionnaire

Notes and instructions on completing the questionnaire:

This short questionnaire assesses some global affairs. We need this information to determine the results to interpret correctly. The information we collect in this questionnaire are only at for the purpose of scientific research into the metabolism and excretion of deoxynivalenol and its glucoside in humans and will be treated confidentially. This means that only the research team which is concerned with the study access to information and should not be passed to third person.

Moreover, everything is encrypted and you never mention your name. Please fill in this questionnaire them to bring along with your urine samples and give it away to one of our employees on a pre-arranged time.

Id number: ……………………………..

What is your date of birth? ……………………..

What is your gender?

☐ Male

☐ Female

What is your height? …………………………………cm

What is your weight? ………………………………..kg

Do you smoke cigarettes, cigars or a pipe?

☐ Yes

☐ No

Do you have a disease relating to ...?

☐ Liver

☐ Kidney

☐ Bile

What drugs and / or supplements you have taken the past month? What dose?

Drugs / supplements	Doses

Are you pregnant or is there a chance that you are pregnant?

☐ Yes

☐ No

Breast-feeding?

☐ Yes

☐ No

Currently follow a diet where some foods should not be eaten because it of illness, allergy or for no reason?

- ☐ Gluten free
- ☐ Slimming free
- ☐ Diabetes
- ☐ Low salt
- ☐ Other……

What diet do you follow?

- ☐ Western European
- ☐ Mediterranean
- ☐ Eastern
- ☐ Halal
- ☐ Kosher
- ☐ Low meat
- ☐ Vegetarian
- ☐ Vegan
- ☐ Other ....

Do you drink coffee every day? If so, how much?

- ☐ No
- ☐ Yes, I drink ……………………………… cups of coffee per day.

Figure 4. Flow diagram with the timing assessments.

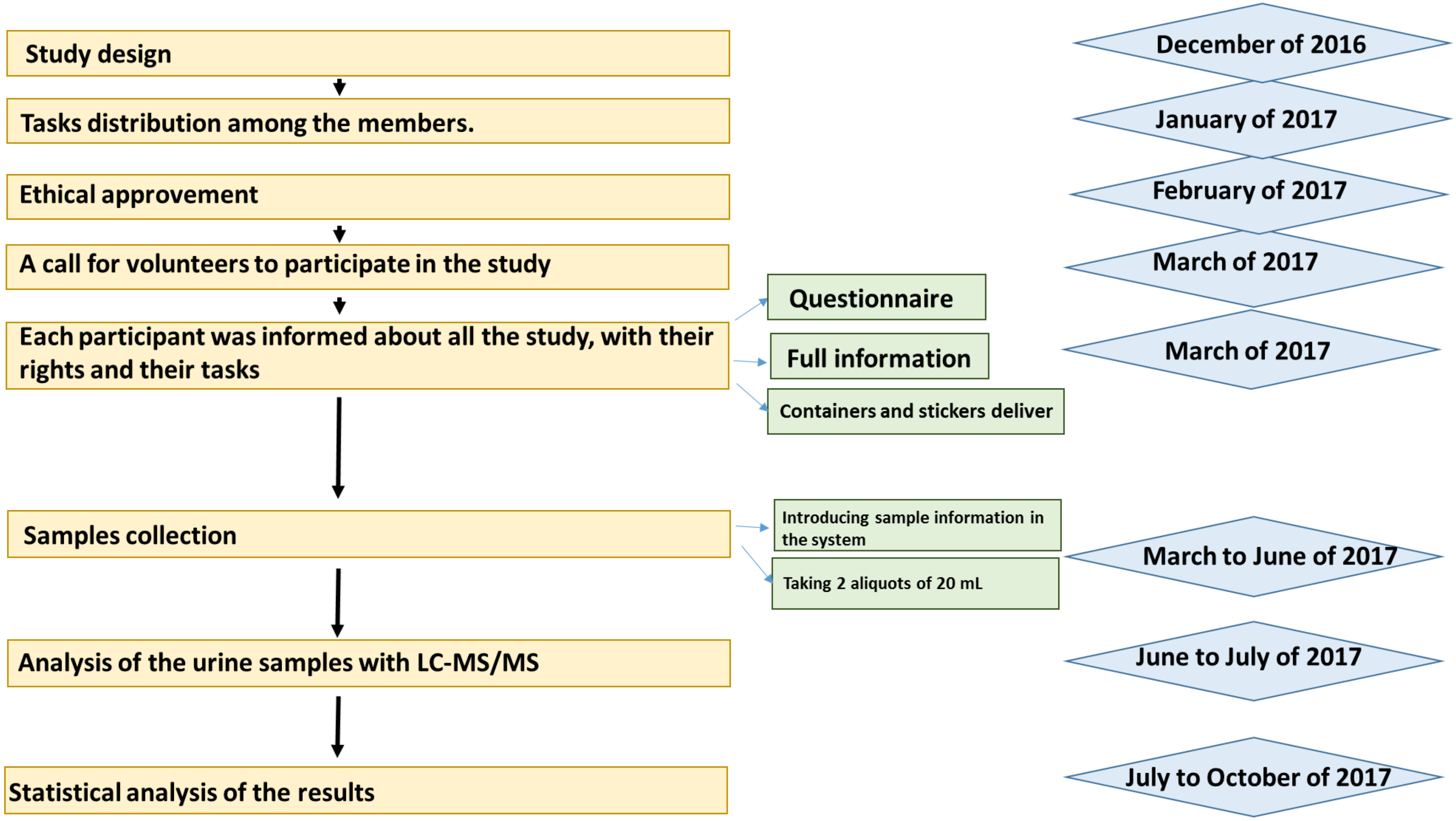

Safety Considerations

The safety of research participants is foremost. Safety aspects of the research should always be kept in mind and information provided in the protocol on how the safety of research participants will be ensured. This can include procedures for recording and reporting adverse events and their follow-up, for example. It is useful to remember that even administering a research questionnaire can have adverse effects on individuals.

The volunteers are cleared informed of the safety procedures followed during the study by letter. Moreover, each participant is informed on his/her right to withdraw from the study at any time and to consult a doctor immediately and inform us if they fell not right during the study. Besides, no medical examinations or interventions are carried out in this study and the toxins are administrated with a water solution. Also we inform that we subministred the TDI level of mycotoxin which cannot cause acute adverse effects.

Follow-Up

The research protocol must give a clear indication of what follow up will be provided to the research participants and for how long. This may include a follow u, especially for adverse events, even after data collection for the research study is completed.

We provide a clear protocol for each volunteer and the instruction to follow during the intervention study.

INSTRUCTIONS FOR VOLUNTEERS:

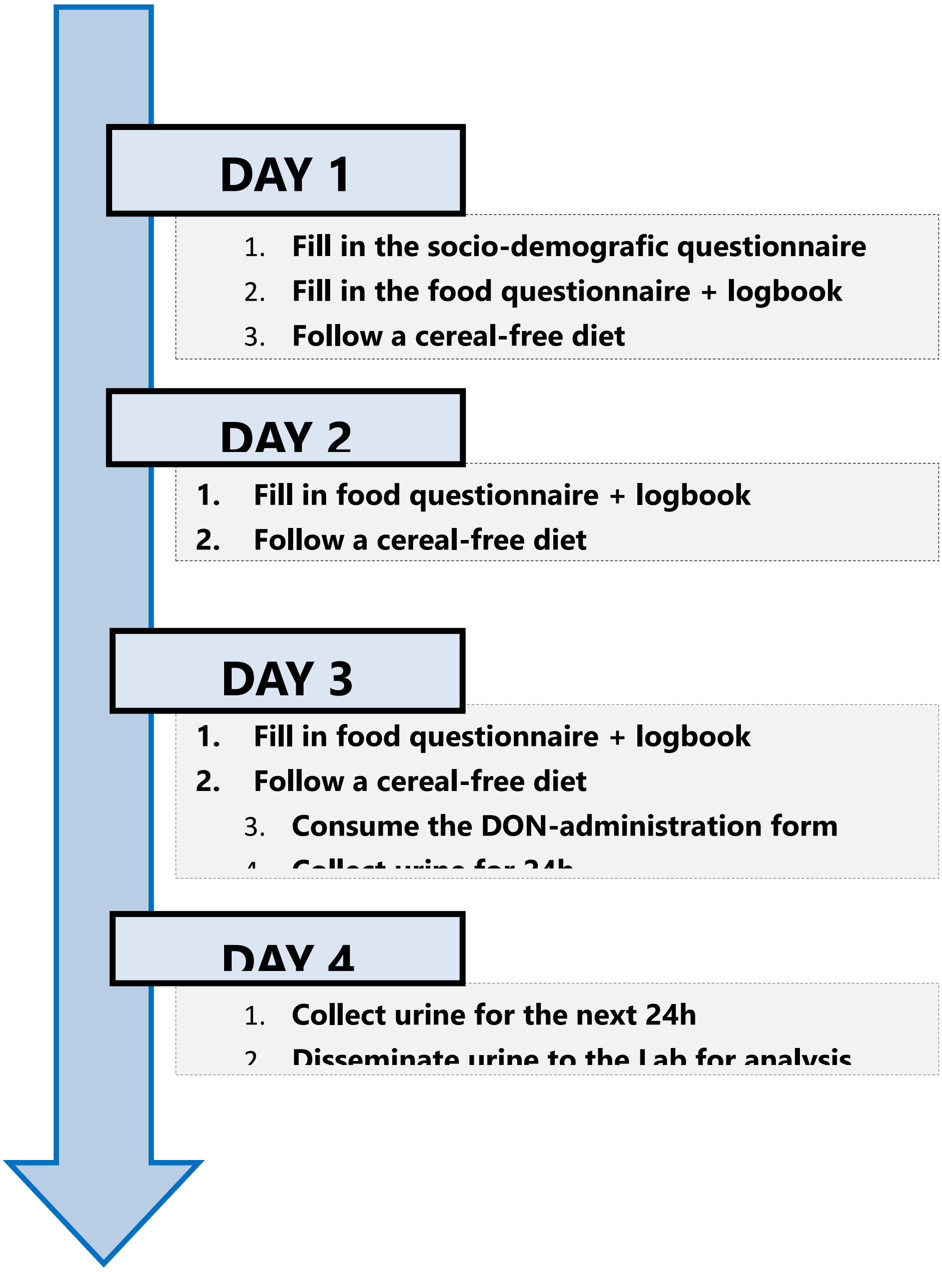

URINE COLLECTION

When getting up on day 3, take your urine (eg 7:00). Record this time on 'your' schedule '. Then you have to collect all your urine for 24 hours (day and night) in different urine containers. Just before the end of the 24 hours, one day later (day 4) on the same time (eg day 4 at 7:00), you catch your urine for the last time. Put an alarm clock so you do not forget about this time to urinate. After this your 24 hour urine is complete. Note the schedule always the time you have all your urine samples collects.

- You can get the urine first in the collector cup (1000 ml graduated cup).

- Then read the amount of urine and record this volume on your schedule.
- Transfer your urine into the smaller urine containers.
- Always use another container per splash.
- On your schedule, indicate which containers were used at any place.
- Keep your urine in a cool dark place in the zipper bag you received (frigo or freezer).
- Keep in mind that small amounts of urine and urine must be collected before or after the stool.
- The collector can be rinsed with hot water (no detergent) and allow to dry in the air.
- Secure a safety pin to your underwear. This reminds you to get your urine all day long
- catching in the container.
- To get enough urine for this study, it is important that you drink enough (2 liters of moisture) for 24 hours).

Data Management and Statistical Analysis

The protocol should provide information on how the data will be managed, including data handling and coding for computer analysis, monitoring and verification. The statistical methods proposed to be used for the analysis of data should be clearly outlined, including reasons for the sample size selected, power of the study, level of significance to be used, procedures for accounting for any missing or spurious data etc. For projects involving qualitative approaches, specify in sufficient detail how the data will be analysed.

Firstly, we agree a coding system for the samples. The coding system is that all samples have two number separated by a "." where the first number is the volunteer id and the second number is the number of the sample of each volunteer. So the first urine sample of the volunteer 1 is 1.1 and the second sample of the volunteer 1 was 1.2. On the other hand, the second sample for the volunteer 2 is 2.1 and the second samples of the volunteer 2 is 2.2. We introduce an excel file all the information for each volunteer id.

Once we analyse the samples, the data of each sample is introduced in the same excel file and the results of each mycotoxin are introduced separately. Then, all obtained results are carried out on molar basis taking the molecular weight of the analytes (DON, 296 g/mole; DON-3-glucoside, 458 g/mole; DON-3-glucuronide and DON-15-glucuronide, 472 g/mole; DOM-1, 280 g/mole; 3-ADON and 15-ADON, 338 g/mole) and the total volume of each sampling point into account. Associations between independent and dependent variables are assessed by using univariate and multivariable linear regression models. Independent variables include age (years), gender, body mass index (BMI),

and the consumption of coffee. The statistical analysis is performed using the software Microsoft Excel® 2010 and SPSS® 15.0.
The excretion profiles are based in values obtained each hour after the bolus administration from a varying number of volunteers per time period. For instance, results from 61 to 120 minutes are clustered in the second hour.

Quality Assurance

The protocol should describe the quality control and quality assurance system for the conduct of the study, including GCP, follow up by clinical monitors, DSMB, data management etc.

The human controlled intervention study is conducted according to the guidelines laid down in the declaration of Helsinki. The standard administration is prepared with commercial standard and the commercial company provided us the quality certification. Moreover, several toxin analysis in the bolus administration are performed before the toxin administration.
Internal standard is used in all the samples. The internal standard offers a correction for the loss of analyte during sample preparation. Moreover, a calibration curve is made for every 40 samples to correct possible variations among different analysis days.

Expected Outcomes of the Study

The protocol should indicate how the study will contribute to advancement of knowledge, how the results will be utilized, not only in publications but also how they will likely affect health care, health systems, or health policies.

The results will be published in a scientific journal and presented in international conferences. The expected outcomes should help to provide a better knowledge in the toxicokinetics of DON and DON-3-glucoside and its renal excretion in humans. Furthermore, results will also give information about which is the best method to analyse DON exposure through the urine. The results will give information about DON biomarkers, excretion rate and speed of excretion to know if it is better to collect only the morning urine or 24 h urine collection.

Dissemination of Results and Publication Policy

The protocol should specify not only dissemination of results in the scientific media, but also to the community and/ or the participants, and consider dissemination to the policy makers where relevant. Publication policy should be clearly discussed- for example who will take the lead in publication and who will be acknowledged in publications, etc.

The results will be published in the scientific journal and we will spread the results in international conferences through oral presentations and posters. We will also try to disseminate the results to the policy makers and policy makers organizations as European union

The dissemination of results will acknowledge JPI Food Biomarkers Alliance (FOODBALL) project (G0D4615N) who financed the study.

Duration of the Project

The protocol should specify the time that each phase of the project is likely to take, along with a detailed month by month timeline for each activity to be undertaken.

The different members of the study started to plan the study design at December of 2016.
On January 2017, the study was fully designed and the tasks were distributed among the members.
On February 2017, the ethical approval was accepted according to the guidelines laid down in the declaration of Helsinki and was approved by the Ethical Committee of the Ghent University Hospital.
On March, a call for volunteers to participate in the study was made. Each participant was informed about their right to withdraw from the study at any time without any reason. It is important to note that no medical examinations were carried out in this study.
From March to June, the samples were collected from the volunteers.
From April to July, the urine samples were analysed by LC-MS/MS
From July to now, the data is being statistically analysed and the article is being written.
The article will be submitted during the autumn of 2017.

Problems Anticipated

This section should discuss the difficulties that the investigators anticipate in successfully completing their projects within the time frame stipulated and the funding requested. It should also offer possible solutions to deal with these difficulties.

Some volunteers could do mistakes during the intervention study and they could eat food which is forbidden during the intervention diet. So, we stipulated that if volunteers are aware that they did a mistake during the intervention diet they have to start again the intervention diet.
Regarding analysis difficulties, mycotoxins concentrations in the urine could be very low so we planned to concentrate the samples if we are not able to detect mycotoxins in the analysed urines.
If some volunteers miss a sample they have to start again the intervention study.

Project Management

This section should describe the role and responsibility of each member of the team

Prof. Sarah de Saeger planned and organized the study. She also looked for the necessary funds to do the intervention study.

Dra. Marthe de Boevre planned and organized the study. She asked for the ethical approvement as well as the insurance. She detailed the research protocol to be followed by the volunteers.

Dr. Arnau Vidal collected the urine samples and did the codification of the samples. He analyses the samples as well as the statistical study of the results.

Ethics

The protocol should have a description of ethical considerations relating to the study. This should not be limited to providing information on how or from whom the ethics approval will be taken, but this section should document the issues that are likely to raise ethical concerns. It should also describe how the investigator(s) plan to obtain informed consent from the research participants (the informed consent process).

The study includes an administration of toxin, however, DON, the studied toxin, is a highly common mycotoxin in cereals. Even, several studies showed that a high percentage of population could exceed the tolerable daily intake of this mycotoxin (Heyndrickx et al., 2015; Vidal et al., 2016). Although acute exposure of animals to DON results in decreased feed consumption (anorexia), vomiting (emesis), abdominal pain, dizziness, headache, throat irritation, diarrhoea, and blood in stool (Rotter et al., 1996). While longer exposure causes reduced growth, and adverse effects on the thymus, spleen, heart, and liver (Sobrova et al., 2010). DON is not a carcinogenic mycotoxin as other mycotoxins like aflatoxins or ochratoxin A and The IARC classified DON in 1993 in Category 3, i.e., not classifiable as to its carcinogenicity to humans. So, although volunteers have to take a toxin administration they are three days without eating cereals which remove the DON exposure for three days. Besides, the DON administration is at TDI level (1 µg/kg/day) and The minimum emetic dose in pigs was 0.05 - 0.2 mg/kg b.w., when given orally.

To obtain the informed consent from the research participants we planned to explain carefully and in great detail all scientific data related to DON. Moreover, some participants are laboratory workers which have a better knowledge of mycotoxins.

Informed Consent Forms

The approved version of the protocol must have copies of informed consent forms (ICF), both in English and the local language in which they are going to be administered. However translations may be carried out after the English language ICF(s) have been approved by the ERC. If the research involves more than one group of individuals, for example healthcare users and healthcare providers, a separate specifically tailored informed consent form must be included for each group. This ensures that each group of participants will get the information they need to make an informed decision. For the same reason, each new intervention also

requires a separate informed consent form. For guidance on how to write an informed consent form, click here**.**

GHENT UNIVERSITY

Toestemmingsformulier interventioneel onderzoek naar de metabolisatie en renale excretie van deoxynivalenol en zijn glucoside bij mensen

Dit wetenschappelijk onderzoek bestudeert hoe het mycotoxine deoxynivalenol en zijn metaboliet, deoxynivalenol-3-glucoside worden gemetaboliseerd en renaal worden geëxcreteerd door het menselijk lichaam na inname. Hiervoor wordt de concentratie van deoxynivalenol en zijn metabolieten in urine onderzocht, alsook de relatie hiervan met de inname van de twee componenten via een capsule enerzijds en natuurlijk gecontamineerde voeding anderzijds. Aan de hand van dit document vragen we uw goedkeuring voor uw deelname aan deze studie.

- Ik ben ervan op de hoogte dat mijn deelname aan deze studie volledig vrijwillig is. Ik kan me op elk ogenblik terugtrekken uit deze studie zonder een reden voor deze beslissing op te geven. De keuze niet deel te nemen of mij terug te trekken uit deze studie houdt geen nadelen in voor mij.
- Ik heb de informatiebrochure gelezen en via deze weg informatie gekregen over de aard, het doel, de duur van de studie en over wat men van mij verwacht. Indien ik verdere inlichtingen wens, weet ik bij wie ik terecht kan.
- Ik ben me ervan bewust dat deze studie ter beoordeling aan de Commissie voor Medische Ethiek van het Universitair Ziekenhuis Gent werd voorgelegd en goedgekeurd. Deze goedkeuring was in geen geval de aanzet om te beslissen om deel te nemen aan deze studie.
- Ik begrijp dat de onderzoekers en de Commissie voor Medische Ethiek mijn gegevens willen inspecteren om de verzamelde informatie te controleren en te verwerken. Door dit document te ondertekenen, geef ik toestemming voor deze controle. Mijn gegevens zullen wel altijd gecodeerd doorgegeven worden, waarbij mijn naam geheim blijft.
- Ik geef de toestemming om mijn urinestaal te bewaren voor later onderzoek. De urinestalen zullen enkel gebruikt worden voor de verdere ontwikkeling en verbetering van wetenschappelijk onderzoek naar deoxynivalenol, alsook om de kennis rond (nieuwe) schimmelmetabolieten, hun inname en de voedselveiligheid te verruimen.

Ik heb dit document volledig gelezen en ik werd voldoende geïnformeerd over de studie. Ik stem in met de inhoud van het document en ik ga akkoord met mijn deelname aan de hierboven beschreven studie.

Een kopie van het ondertekende toestemmingsformulier zal naar u worden toegestuurd.

Naam vrijwilliger:……………………………………………………………………………………………

Contactgegevens (emailadres of telefoonnummer): ……………………………………………………

Datum: ……………………………………………………………………………………………………

Handtekening:……………………………………………………………………………………………

Toestemmingsformulier dd 21/11/2016

Ik bevestig dat ik de aard, het doel, en de te voorziene effecten van de studie heb uitgelegd aan de bovenvermelde vrijwilliger.

De vrijwilliger stemde toe om deel te nemen door zijn/haar persoonlijk gedateerde handtekening te plaatsen.

Naam van de persoon
die voorafgaande uitleg
heeft gegeven: ____________________________________

Datum: ____________________________________

Handtekening:

GHENT UNIVERSITY

Authorization form interventional study on the metabolization and renal excretion of deoxynivalenol and its glucoside in humans.

This scientific research studies how mycotoxin deoxynivalenol and its metabolite, deoxynivalenol-3-glucoside are metabolized and excreted renally by human body after ingestion. For this, the concentration of deoxynivalenol and its metabolites is in urine examined as well as its relation to the intake of the two components via a capsule on the one hand and naturally contaminated food on the other. Based on this document, we ask for your approval for your participation in this study.

- I am aware that my participation in this study is fully voluntary. I can handle myself at any time withdraw from this study without giving a reason for this decision. The choice is not part of take or withdraw me from this study does not harm me.

- I have read the information brochure and received information about the nature, the purpose, the duration of this road of the study and about what I expect from me. If I wish further information, I know who I am can rightly

- I am aware that this study is being reviewed by the Commission for Medical Ethics University Hospital Ghent was submitted and approved. This approval was by no means the to decide to participate in this study.

- I understand that the researchers and the Commission for Medical Ethics want to inspect my data to check and process the collected information. By signing this document, I give permission for this check. My data will always be encrypted, with my name remains secret.

- I give permission to keep my urine sample for later examination. The urine samples will only used for further development and improvement of scientific research for deoxynivalenol, as well as the knowledge about (new) fungal metabolites, their intake and the to expand food safety.

I have read this document fully and I was adequately informed about the study. I agree with the content of the document and I agree with my participation in the study described above.

A copy of the signed consent form will be sent to you.

Volunteer Name: ....

Contact details (email address or telephone number): ....

Date: ....

Signature:...

I confirm that I have explained the nature, purpose and the foreseeable effects of the study to the volunteer mentioned above.

The volunteer agreed to participate by placing his / her personally dated signature.

Name of the person the previous explanation has given: __________________

Date: ____

Signature:

Other support for the Project

This section should provide information about the funding received or anticipated for this project from other funding organizations.

Only, FOODBALL project with FWO gave funds for this project. 50000 €

Collaboration with other scientists or research institutions

Links to other projects

Curriculum Vitae of investigators

The CV of the Principal investigator and each co-investigators should be provided. In general each CV should not be more than one page, unless a complete CV is specifically requested for.

**Name:** De Saeger Sarah<br>**Date of Birth:** 10/09/1971, Belgium<br>**Position:** Full professor at Ghent University<br>**Education:** Pharmacist, Ghent University, 1994; PhD in Pharmaceutical Sciences, Ghent University, 1999

A. Positions and honours

- **Positions and employment:**

Ghent University, Department of Bioanalysis (Laboratory of Food Analysis) as PhD researcher (1994-1999), Post-doctoral Assistant (1999-2005), Assistant Professor (2005-2010), Associate Professor (2010-2014) and Full Professor (2014-now).

- **Teaching experience:**

Professor Sarah De Saeger teaches (food-related) courses at the Faculty of Pharmaceutical Sciences: Bromatology, Special Nutrition, Bioanalytical practical; in the past she was also teaching Diet products and Food safety.
She was involved in educational reform projects: bioanalytical practical (2012), bromatology in the community pharmacy (2014), IbeS: innovation by e-simulation (2014).

Related to developmental cooperation:
She coordinated the VLIR-UOS Short Training Initiative 'Intensive Training on Mycotoxin Analysis' in 2012, 2013 en 2014 for trainees from developing countries.

- **Research experience:**

Research topics: food safety, food analysis
Expertise: mycotoxins
Coordinator of the association research platform MYTOX (www.mytox.be).

Related to developmental cooperation:
Coordinator of the international thematic network MYTOX-SOUTH (mytoxsouth.org) which is currently focused on Africa.
Established the Joint Laboratory of Mycotoxin Research of the Ghent University-Shanghai Jiao Tong University-Chinese Academy of Sciences (Shanghai Institutes of Biological Sciences).

- **Professional memberships:**

At Ghent University:
Faculty Representative at the Research Council (2013-…), IOF-council (2013-…), Committee Development Cooperation (2012-…), substitute Board of Governors (2014-…), steering committee China platform (2013-…), steering committee Russia platform (2016-…)

External:
Member of the Executive Committee of the International Society for Mycotoxicology (ISM) (Secretary: 2017-2020).
Member of the Scientific Committee of the Belgian Federal Agency for the Safety of the Food Chain (FASFC) (2015-…)
Member of the European Food Safety Authority (EFSA) CONTAM working group on *Aspergillus* toxins (18/03/2011-06/2013) and *Fusarium* toxins (2014- …)

- **Awards**

2015 Ghent University Prometheus Award for Research
2015 International DBN Science & Technology Award registered by the Ministry of Science and Technology, China.

B. Publications and research projects

Publications: 328 publications including 221 peer reviewed papers and editor of two books (https://biblio.ugent.be/person/801000957125); 1 patent and 1 patent application.
Promotor of 18 defended Phds.
Research projects: many EU (FP7 and H2020), EFSA, FAO/WHO, VLIR-UOS, HERCULES, FWO, IWT, FOD Volksgezondheid, BELSPO, and BOF-projects.

Best 5 selected peer-reviewed scientific publications, with relevance to developmental cooperation:

- Hove, M., De Boevre, M., Lachat, C., Jacxsens, L., Nyanga, L.K., De Saeger, S. 2016. Occurrence and risk assessment of mycotoxins in subsistence farmed maize from Zimbabwe. FOOD CONTROL, 69 36-44.
- Njumbe Ediage, E., Van Poucke, C. & De Saeger, S. 2015. A multi-analyte LC-MS/MS method for the analysis of 23 mycotoxins in different sorghum varieties : the forgotten sample matrix. FOOD CHEMISTRY, 177 397-404.

- Matumba, L., Van Poucke, C., Njumbe Ediage, E., Jacobs, B. & De Saeger, S. 2015. Effectiveness of hand sorting, flotation/washing, dehulling and combinations thereof on the decontamination of mycotoxin-contaminated white maize. FOOD ADDITIVES AND CONTAMINANTS PART A-CHEMISTRY ANALYSIS CONTROL EXPOSURE & RISK ASSESSMENT, 32(6) 960-969.
- Yogendrarajah P., Jacxsens L., Lachat C., Walpita C.N., Kolsteren P., De Saeger S., De Meulenaer B.2014. Public health risk associated with the co-occurrence of mycotoxins in spices consumed in Sri Lanka. Food and Chemical Toxicology 74 : 240-248.
- Njumbe Ediage E., Diana Di Mavungu J., Song S., Sioen I., De Saeger S.2013. Multi mycotoxin analysis in urines to assess infant exposure: a case study in Cameroon. Environment International 57-58 : 50-59.

Name: De Boevre Marthe
Date of Birth: 07/02/1986, Belgium
Position: Post-doctoral Assistant at Ghent University
Education: Pharmacist, Ghent University, 2009; PhD in Pharmaceutical Sciences, Ghent University, 2013

A. Positions and honours

- **Positions and employment:**

Ghent University, Department of Bioanalysis (Laboratory of Food Analysis) as PhD researcher (2009-2013), Post-doctoral Assistant (2013- to date).

- **Teaching experience:**

Dr. Marthe De Boevre is co-teaching (with Prof. Sarah De Saeger) (food-related) courses at the Faculty of Pharmaceutical Sciences: Bromatology, Special Nutrition and Bioanalytical practical. She is coordinator of the Bioanalytical practical.
She was involved in educational reform projects: bioanalytical practical (2012), bromatology in the community pharmacy (2014) and IbeS: innovation by e-simulation (2014).

Related to developmental cooperation:
She was member of the Organizing Committee of the VLIR-UOS Short Training Initiative 'Intensive Training on Mycotoxin Analysis' in 2012, 2013 en 2014 for trainees from developing countries.

- **Research experience:**

Research topics: food safety, food analysis, human health, risk assessment
Expertise: mycotoxins
Secretary of the association research platform MYTOX (www.mytox.be).

Related to developmental cooperation:
Coordinator of the international thematic network MYTOX-SOUTH (mytoxsouth.org) which is currently focused on Africa.
Member of the Joint Laboratory of Mycotoxin Research of the Ghent University-Shanghai Jiao Tong University-Chinese Academy of Sciences (Shanghai Institutes of Biological Sciences).

- **Professional memberships:**

At Ghent University:
Representative of the Academic Staff (gamma) at the Research Council (2015- to date), Member of the 'Commission of Internationalization' at the Faculty of Pharmaceutical Sciences, UGent.
Editor of World Mycotoxin Journal.

- **Awards**

2016 - International Congress Mycotoxins and Cancer, Marrakesh (Marocco) - 24-25/03/2016, Impact of chronic multi-mycotoxin exposure in Europe on cancer incidence: a basis to develop future public health strategies – best poster award
2013 - Most cited publication of 2013 in Food Additives and Contaminants part A (Q1 – 32/124, IF 2.220). Development and validation of an LC-MS/MS method for the simultaneous determination of

deoxynivalenol, zearalenone, T-2-toxin and some masked metabolites in different cereals and cereal-derived food (29 (5), 819-835 (2012))

B. Publications and research projects

Publications: 37 publications including peer reviewed papers and editor of a Special Issue (Toxins) (https://biblio.ugent.be/person/002004253204)
Promotor of 5 Phds, Melody Hove, Cynthia Chilaka, Karl De Ruyck, Mohamed Fathi Abdallah & Victor Kagot.
Research projects: many EU (H2020), EFSA, FAO/WHO, VLIR-UOS, HERCULES, FWO, IWT, FOD Volksgezondheid, BELSPO, and BOF-projects.

Best 5 selected peer-reviewed scientific publications, with relevance to developmental cooperation:

- M. Hove, M. De Boevre, C. Lachat, L. Jacxsens, L. Nyanga, S. De Saeger; Food Control (69, 36-44) (*Q1 – 18/124, IF 2.738)* (2016). Occurrence and Risk Assessment of Mycotoxins in Subsistence Farmed Maize from Zimbabwe.
- C. Adaku Chilaka, M. De Boevre, O. Atanda and S. De Saeger; Food Control (69, 36-44) (*Q1 – 18/124, IF 2.738)* (accepted, 2017). Prevalence of *Fusarium* mycotoxins in cassava and yam products from some selected Nigerian markets
- A. Ayelign, A. Zewdu Woldegiorgis, A. Adish, M. De Boevre, E. Heyndrickx and S. De Saeger; Food Additives & Contaminants: Part A (DOI 10.1080/19440049.2017.1350290) (*Q1 – 32/124, IF 2.220*) Assessment of aflatoxin exposure among young children in Ethiopia using urinary biomarkers.
- C. Adaku Chilaka, M. De Boevre, O. Atanda and S. De Saeger; Toxins (9,1, 19) (*Q1 – 16/89, IF 3.9*) Status of *Fusarium* mycotoxins in sub-Saharan Africa: a review on emerging trends and mitigation strategies towards food control.
- M. Hove, A. Vidal Corominas, M. De Boevre, LK Nyanga, S. De Saeger 2017 Human dietary exposure to aflatoxins and fumonisins and related risk assessment: A focus on subsistence farming populations of Zimbabwe (Food and Chemical Toxicology, under review, final revision).

Name: Vidal Corominas, Arnau
Date of Birth: 27/03/198, Spain
Position: Post-doctoral Assistant at Ghent University
Education: Agricultural Engineer, Lleida University, 2012; PhD in Agrofood techonologies, Lleida University, 2017

A. Positions and honours

- **Positions and employment:**

Ghent University, Department of Bioanalysis (Laboratory of Food Analysis) as Post-doctoral Assistant (2017- to date).

- **Teaching experience:**

Teacher in Food fermentations in degree of science and technology of foods in the Food technology degree in the University of Lleida. (2016).
Supervisor of several degree and master thesis in the University of Lleida and University of Ghent
Research topics: food safety, food analysis, human health, risk assessment
Expertise: mycotoxins

Related to developmental cooperation:
Operation manager of the international thematic network MYTOX-SOUTH (mytoxsouth.org) which is currently focused on Africa.

- **Awards**

2015 - National Congress Mycotoxins, Valencia (Spain) - 25-26/06/2015, Stability of deoxynivalenol and ochratoxin A during the breadmaking process – best oral presentation award

B. Publications and research projects

Publications: 12 publications including peer reviewed.

Best 5 selected peer-reviewed scientific publications, with relevance to developmental cooperation:

- A. Vidal, S. Marín, A.J. Ramos, G. Cano-Sancho, V. Sanchis. Food and Chemical Toxicology (53, 133-138) (Q1-8/128, IF 3.78) (2013). Determination of aflatoxins, deoxynivalenol, ochratoxin A and zearalenone in wheat and oat based bran supplements sold in the Spanish market.
- A. Vidal, V. Sanchis, A.J. Ramos, S. Marín. Food Chemistry (178, 276-286) (Q1-12/128, IF 4.53) (2015). Thermal stability and kinetics of degradation of deoxynivalenol, deoxynivalenol conjugates and ochratoxin A during baking of wheat bakery products.
- A. Vidal, A. Ambrosio, V. Sanchis, A.J. Ramos, S. Marín. Food Chemistry (208, 288-296) (Q1-12/128, IF 4.53) (2016). Enzyme bread improvers affect the stability of deoxynivalenol and deoxynivalenol-3-glucoside during breadmaking.
- A. Vidal, G. Cano-Sancho, S. Marín, A. J. Ramos, V. Sanchis. World Mycotoxin Journal (9, 597-612) (*Q2 – 37/129, IF 2.17)* (2016). Multidetection of urinary ochratoxin A, deoxynivalenol and its metabolites: pilot time-course study and risk assessment in Catalonia, Spain.
- M. Hove, A. Vidal Corominas, M. De Boevre, LK Nyanga, S. De Saeger 2017 Human dietary exposure to aflatoxins and fumonisins and related risk assessment: A focus on subsistence farming populations of Zimbabwe (Food and Chemical Toxicology, under review, final revision).

Other research activities of the investigators

The Principal investigator should list all current research projects that he/she is involved in, the source of funding of those projects, the duration of those projects and the percentage of time spent on each.

Dr. Arnau Vidal is only in the FOODBALL project. He spent full time in this project and the duration of the project is one year, from January 2017 to January 2018.

Financing and Insurance

The insurance:

Clinical Trials
Certificate of Insurance

Allianz Global Corporate & Specialty SE
Belgium Branch
Uitbreidingstraat 86
2600 Berchem
Belgium

Date:	08.02.2017
Policy Number:	BEL000862
Policy Inception Date:	01/01/2017
Policy Expiry Date:	31/12/2017

This certifies that Allianz Global Corporate & Specialty SE (Belgium Branch) insures the Clinical Trial listed below in accordance with the terms and conditions of the above referenced Policy. This certificate of insurance neither affirmatively nor negatively amends, extends nor alters the coverage afforded by the policy numbered in this certificate. This certificate is issued in respect of the Belgian Law of May 7, 2004 concerning the Clinical Trials.

Insured:	Universiteit Gent<br>Sint-Pietersnieuwstraat 25<br>9000 Gent<br>Belgium
Insured experiment:	All trials started, meaning approved by the Ethics Committee, in between the policy insurance period are covered for their entire duration as shown in the respective Protocols
Territoriality:	Belgium
Extended reporting period:	36 months starting at the expiry date of each trial
Insured limits of indemnity:	EUR 750.000 per tested person limited to EUR 2.500.000 per trial and EUR 7.500.000 per year for bodily injury and property damage combined.

Signed by: Kristel De Jonghe, Liability Manager

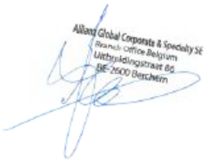

References:

Pestka, J. J. Deoxynivalenol: mechanisms of action, human exposure, and toxicological relevance. *Archives of Toxicology* **84**, 663-679, doi:10.1007/s00204-010-0579-8 (2010).

De Boevre, M. *et al.* Development and validation of an LC-MS/MS method for the simultaneous determination of deoxynivalenol, zearalenone, T-2-toxin and some masked metabolites in different cereals and cereal-derived food. *Food Additives and Contaminants* **29**, 819-835 (2012).

Marin, S., *et al*. Mycotoxins: Occurrence, toxicology, and exposure assessment. *Food and Chemical Toxicology* **60**, 218-237 (2013).

Heyndrickx, E. *et al.* Human biomonitoring of multiple mycotoxins in the Belgian population: Results of the BIOMYCO study. *Environment International* **84**, 82-89 (2015).

Vidal, A. et al. Multidetection of urinary ochratoxin A, deoxynivalenol and its metabolites: pilot time-course study and risk assessment in Catalonia, Spain. *World Mycotoxin Journal* **9**, 597-612, (2016).

Berthiller, F., Schuhmacher, R., Adam, G. & Krska, R. Formation, determination and significance of masked and other conjugated mycotoxins. *Analytical and Bioanalytical Chemistry* **395**, 1243-1252 (2009).

Rychlik, M. et al. Proposal of a comprehensive definition of modified and other forms of mycotoxins including "masked" mycotoxins. *Mycotoxin Research* **30**, 197-205 (2014).

Broekaert, N. et al. Oral Bioavailability, Hydrolysis, and Comparative Toxicokinetics of 3-Acetyldeoxynivalenol and 15-Acetyldeoxynivalenol in Broiler Chickens and Pigs. *Journal of Agricultural and Food Chemistry* **63**, 8734-8742, (2015).

Grabley, S., Gareis, M., Bockers, W. & Thiem, J. Glycosylation of mycotoxins. Synthesis-Stuttgart, 1078-1080 (1992).

Nagl, V. *et al.* Metabolism of the masked mycotoxin deoxynivalenol-3-glucoside in pigs. *Toxicology Letters* **229**, 190-197 (2014).

De Boevre, M. *et al.* Natural occurrence of mycotoxins and their masked forms in food and feed products. *World Mycotoxin Journal* **5**, 207-219, doi:10.3920/WMJ2012.1410 (2012).

Berthiller, F. *et al.* Hydrolytic fate of deoxynivalenol-3-glucoside during digestion. *Toxicology Letters* **206**, 264-267 (2011).

Versilovskis, A. *et al.* Simultaneous determination of masked forms of deoxynivalenol and zearalenone after oral dosing in rats by LC-MS/MS. *World Mycotoxin Journal* **5**, 303-318 (2012).

JECFA. WHO Technical Report Series 959: JECFA 2010: Seventy-second report of the Joint FAO/WHO Expert Committee on Food Additives: evaluation of certain contaminants in food, 2010. *WHO Technical Report Series 959* (2010).

Warth, B. *et al.* Assessment of human deoxynivalenol exposure using an LC-MS/MS based biomarker method. *Toxicology Letters* **211**, 85-90 (2012).

Turner, P. C. *et al.* in *Food Additives & Contaminants: Part A* Vol. 25 864-871 (Taylor & Francis, 2008).

Warth, B., Sulyok, M., Berthiller, F., Schuhmacher, R. & Krska, R. New insights into the human metabolism of the Fusarium mycotoxins deoxynivalenol and zearalenone. *Toxicology Letters* **220**, 88-94 (2013).

Clewell, H. J., Tan, Y. M., Campbell, J. L. & Andersen, M. E. Quantitative interpretation of human biomonitoring data. *Toxicology and Applied Pharmacology* **231**, 122-133 (2008).

Rotter, B.A., Prelusky, D.B., Thompson, B.K. The role of tryptophan in DON-induced feed rejection. *Journal of Environmental Science and Health - Part B Pesticides, Food Contaminants, and Agricultural Wastes* **31**, 1279-1288 (1996).

Sobrova, P. et al. Deoxynivalenol and its toxicity. *Interdisciplinary Toxicology* **3**, 94-99 (2010).
